# The phylotranscriptomic profile of angiosperm seed development follows a reverse hourglass pattern

**DOI:** 10.1093/plcell/koaf266

**Published:** 2025-11-12

**Authors:** Asif Ahmed Sami, Leónie Bentsink, Mariana A S Artur

**Affiliations:** Wageningen Seed Science Center, Laboratory of Plant Physiology, Wageningen University, Wageningen 6708PB, The Netherlands; Wageningen Seed Science Center, Laboratory of Plant Physiology, Wageningen University, Wageningen 6708PB, The Netherlands; Wageningen Seed Science Center, Laboratory of Plant Physiology, Wageningen University, Wageningen 6708PB, The Netherlands

## Abstract

The angiosperm seed life cycle encompasses three broad phases—embryogenesis, maturation, and germination. Seed maturation is particularly critical, bridging embryo development and germination while enabling accumulation of nutrient reserves and acquisition of traits like desiccation tolerance, essential for survival in diverse environments. While embryogenesis and germination in *Arabidopsis thaliana* are known to follow an hourglass-like phylotranscriptomic pattern (with higher gene expression conservation in the mid-stages), the transcriptomic landscape of seed maturation and the complete seed life cycle remain unexplored. Using publicly available RNA-seq data, we generated transcriptome age index and transcriptome divergence index profiles of all three phases of the Arabidopsis seed life cycle, revealing a reverse hourglass-like phylotranscriptome pattern. Seed maturation exhibited increased expression of younger genes with divergent expression patterns compared to embryogenesis and germination, which was conserved in other dicots and monocots. Tissue-specific analyses revealed that, in monocots, the endosperm has increased expression of younger genes during maturation. We found that, similar to pollen development, seed maturation is a pivotal phase enabling the expression of young, rapidly evolving genes. We propose the “out of the seed” hypothesis, where seed maturation serves as a landscape for expressing new genes and promoting functional specialization.

## Teaser

Angiosperm seed development exhibits a reverse phylotranscriptomic pattern, with early and late stages showing greater conservation and mid-phase showing higher molecular divergence.

## Introduction

The evolution of the seed habit is a major innovation in the plant kingdom and is regarded as one of the most successful means of plant sexual reproduction (Haig and Westoby 1989). Seeds have facilitated the large-scale diversification of plants and paved the way for gymnosperms and angiosperms to dominate global ecosystems (Linkies et al. 2010). The seed life cycle can be broadly divided into three phases: embryogenesis, maturation, and germination (Kermode 1990; Holdsworth et al. 1999). Embryogenesis marks a series of coordinated cell divisions that establish the basic body plan of the plant. Subsequently, in the maturation phase, seeds accumulate nutrient reserves (e.g. oils, sugars, and proteins) and acquire traits such as germinability, dormancy, desiccation tolerance, and longevity, which are vital for their survival in diverse environments (Kermode et al. 1986; Kermode 1990). Post-maturation, most seeds enter a quiescent dry state, whereby they can persist in the environment for prolonged periods before conditions are favorable for germination to commence.

On the contrary, in most animals, embryonic development is a continuous process devoid of a quiescent phase. Animal embryogenesis follows an hourglass model of development, whereby embryos of different species exhibit morphological divergence during early and later stages but converge toward higher resemblance during mid-embryonic development (known as the phylotypic stage) (Galis and Metz 2001; Willmore 2012; Irie and Kuratani 2014; Drost et al. 2017). The embryonic hourglass was also substantiated on a transcriptome level using phylotranscriptomic indices (Domazet-Lošo and Tautz 2010; Quint et al. 2012), which link gene evolutionary distance to expression levels to determine the transcriptome age at a specific developmental stage. One such index is the transcriptome age index (TAI), which takes into account the evolutionary age of a gene. A high TAI value is indicative of a younger transcriptome, and a low TAI value suggests an older transcriptome. A counterpart to the TAI is the transcriptome divergence index (TDI), which reflects sequence divergence. A high TDI value is associated with a transcriptome expressing rapidly evolving genes. These indexes have demonstrated the presence of a molecular hourglass signature in animals, plants, fungi, and brown algae in both bulk and single-cell RNA-seq data (Domazet-Lošo and Tautz 2010; Quint et al. 2012; Cheng et al. 2015; Ma and Zheng 2023; Lotharukpong et al. 2024; Wu et al. 2024). The embryonic hourglass has been observed across angiosperms, including *Arabidopsis thaliana* L. (hereafter referred to as Arabidopsis) (Quint et al. 2012) and *Brassica* spp. (Gao et al. 2022), wheat (Xiang et al. 2019), and maize (*Zea mays* L.) (Wu et al. 2024). Remarkably, a similar hourglass pattern was also reported post-embryonically, such as in Arabidopsis seed germination (Drost et al. 2016). The dual hourglass-like patterns during the seed life cycle suggest a higher reliance on evolutionarily conserved genes during these two phases.

Unlike embryogenesis and germination, seed maturation lacks an in-depth phylotranscriptomic study, likely due to prior unavailability of high-resolution temporal RNA-seq of this phase in Arabidopsis (Artur et al. 2024). Consequently, a comprehensive picture of the phylotranscriptomic pattern throughout the entire seed life cycle is missing. Seed maturation bridges embryo development and germination, representing a critical phase in the seed life cycle during which key survival traits (i.e. dormancy, desiccation tolerance, and longevity) are acquired. Because environmental factors directly influence gene expression during this phase (He et al. 2014; Penfield and MacGregor 2017; Bezodis and Penfield 2024), maturation plays a vital role in determining the developmental fitness, plasticity, and ecological success of seeds. Thus, understanding the dynamics and drivers of evolution during phase transitions throughout the seed life cycle can shed light on the ecological importance of physiological adaptations in seeds.

Here, we examined the phylotranscriptome landscape of Arabidopsis seed life cycle to identify the evolutionary patterns across key phase transitions. We reveal that, unlike embryogenesis and germination, the Arabidopsis maturation transcriptome is characterized by high TAI and TDI values, with the phylotranscriptomic pattern of the entire seed life cycle resembling a reverse hourglass. Leveraging available RNA-seq datasets, we generated the first phylotranscriptomic profile of the seed life cycle across multiple angiosperm species, including monocots and dicots. Remarkably, we identified a conserved high TAI pattern during maturation in both lineages, with the endosperm making a significant contribution to the high TAI during the maturation of monocots. Comparison of seed maturation with pollen development, another phase with high TAI and a reverse hourglass pattern (Wu et al. 2014; Cui et al. 2015; Gossmann et al. 2016; Julca et al. 2021), revealed that the younger genes driving the reverse hourglass pattern are unique to the seed transcriptome. Overall, our findings establish seed maturation as a pivotal developmental phase enabling the expression of young genes and rapidly evolving genes critical for seeds’ adaptive capacity in their surrounding environment.

## Results

### Transcriptome age and divergence indices reveal a reverse hourglass pattern during the Arabidopsis seed life cycle

The developmental hourglass pattern during embryogenesis and germination is well-documented in Arabidopsis (Quint et al. 2012; Drost et al. 2016). However, to identify the evolutionary patterns across key phase transitions of the entire seed life cycle, a high-resolution temporal transcriptome of seed maturation is essential. We therefore combined recently available Arabidopsis seed maturation RNA-seq data (Artur et al. 2024) with previously generated RNA-seq data of embryogenesis (Hofmann et al. 2019) and germination (Bai et al. 2025). Together, these datasets cover the complete Arabidopsis seed life cycle, from the preglobular embryonic stage to 72 h after imbibition (Supplementary Table S1). Although the datasets covering the three different phases are from different studies, each of the embryogenesis and germination datasets contains at least one stage that belongs to the maturation phase. For instance, the embryogenesis dataset contains the bent cotyledon and mature green stages, which are part of seed maturation, while the germination dataset contains the dry seed stage, marking the end of seed maturation (Baud et al. 2002).

**Figure 1.**
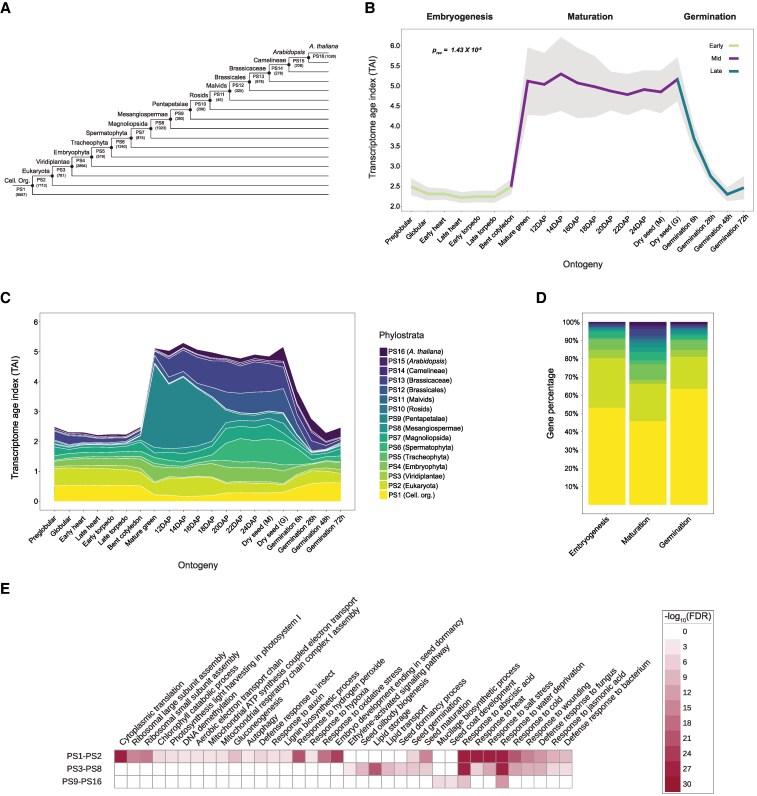
TAI profile throughout the seed life cycle of *Arabidopsis*. **A)** Phylostratigraphy of *Arabidopsis* showing the distribution of protein-coding genes over sixteen PS (PS1–PS16). The number of genes under each phylostratum is indicated within brackets. **B)** TAI pattern during three distinct phases (embryogenesis, maturation, and germination) of the *Arabidopsis* seed life cycle. The stages Preglobular—Mature green are from the Hofmann et al. (2019), 12DAP—Dry seed (M) from Artur et al. (2024), and Dry seed (G) from Bai et al. (2025) dataset. Colored regions in the mean TAI line correspond to stages that belong to each phase. During the reverse hourglass test, these stages corresponding to embryogenesis, maturation, and germination stages were defined as early, mid, and late developmental stages. Shaded regions around the TAI line indicate the standard deviation (s.d.) calculated from 50,000 permutations. The *P*-value indicates the significance of the reverse hourglass test. DAP stands for days after pollination. **C)** Contribution of individual phylostratum to the overall TAI profile. PS are arranged from youngest to oldest from top to bottom. The TAI values are not cumulative, and each band indicates the contribution of a single phylostratum alone. **D)** Percentage of genes from each phylostratum among the top 5% most highly expressed genes for each phase. For each phase, genes were filtered on an average TPM ≥ 1 threshold and then sorted from highest to lowest expression to select the top 5% of genes. **E)** GO terms enriched among the top 5% maturation genes. Genes were grouped into three groups—PS1–PS2, PS3–PS8, and PS9–PS16.

Calculation of phylotranscriptomic age indices requires generating phylostratigraphic age maps of genes. By executing homology searches against 4,940 species (Supplementary Table S2), we classified Arabidopsis genes into different phylostrata (PS) based on their originating nodes. This led to the grouping of all Arabidopsis genes into discrete age groups ranging from PS1 to PS16 (Fig. 1A and Supplementary Table S3), with PS1 representing the oldest and PS16 the youngest phylostratum. The TAI measure relies on the phylogenetic rank of a gene and its expression at a given stage (Domazet-Lošo and Tautz 2010). Here, TAI values were calculated for each stage of the Arabidopsis seed life cycle using the evolutionary age (PS1-PS16) and transcript per million (TPM) expression values of each gene (Supplementary Data S1). A higher or lower TAI value indicates a younger or older transcriptome age, respectively. We visualized the TAI profiles for each phase individually and observed that both embryogenesis and germination displayed a significant hourglass pattern (*p_hg_* = 0.000116 and 0.0159, respectively), with a characteristic phylotypic phase during mid-development (Supplementary Fig. S1A and C) (Quint et al. 2012; Drost et al. 2016). In contrast, the TAI profile of maturation did not deviate significantly from a flat line, indicating little variation in transcriptome age and suggesting a stable transcriptome profile throughout this phase (Supplementary Fig. S1B, *p_flt_* = 0.776). Plotting the TAI values of the three stages sequentially (embryogenesis, maturation, and germination) revealed a strikingly high TAI during seed maturation, distinguishing it from the two adjacent phases (Fig. 1B). This high TAI value was evident for all maturation stages, regardless of the data source. Intriguingly, as opposed to the well-known hourglass pattern of development (high-low-high TAI), the TAI profile of the entire seed life cycle resembled a reverse hourglass pattern (low-high-low TAI) when maturation was considered the mid-developmental phase (Fig. 1B). The pattern showed significance (*p_rev_* = 1.43×10^−6^) when tested with the myTAI package (Drost et al. 2018) against a reverse hourglass pattern. Although less prevalent, such a reverse hourglass pattern has been previously associated with embryonic (and non-embryonic) development, both within a species (Wu et al. 2019) and across species from different phyla (Levin et al. 2016; Leiboff and Hake 2019; Ganofsky et al. 2025). Opposite to the hourglass pattern, the reverse hourglass marks a higher degree of divergence during mid-development (in this case, maturation) compared to early (embryogenesis) and late (germination) developmental stages.

The significance of the reverse hourglass pattern was robust to different RNA-seq data transformation methods (Supplementary Fig. S2). We visualized the RNA-seq samples using principal component analysis (PCA) plots, following two transformations: (i) a log2 transformation on the TPM counts, and (ii) a variance-stabilizing transformation using DESeq2 on the raw read counts. In both cases, the PCA showed that the maturation stages from data from different sources (Hofmann et al. 2019; Artur et al. 2024; Bai et al. 2025) clustered together either on PC1 or on both PC1 and PC2 (Supplementary Fig. S3A and B). This showed that the reverse hourglass pattern is not an artifact of data merging from multiple sources.

While TAI captures ancestral distance-based evolutionary signals, the TDI depicts evolutionary signals from recent sequence divergence (Quint et al. 2012; Drost et al. 2015). We then calculated the TDI per RNA-seq sample by combining Ka/Ks (non-synonymous/synonymous substitutions) ratio of each gene with its expression level (Supplementary Table S4). Consistent with the TAI profile, the TDI profile also resembled a reverse hourglass (*p_rev_* = 0.00783, Supplementary Fig. S1D), with maturation exhibiting higher TDI values than the adjacent phases. Taken together, our results suggest that embryogenesis and germination exhibit relatively lower TAI and TDI values, likely due to greater reliance on more conserved or less rapidly evolving genes (Quint et al. 2012; Drost et al. 2016). On the other hand, the seed maturation exhibits the opposite trend, characterized by a major contribution of younger and more rapidly evolving genes.

### Genes underlying the high maturation TAI are associated with seed maturation traits

The mean relative expression of older PS groups (PS1-PS3) was significantly higher than that of the younger PS groups (PS4-PS16) during embryogenesis. In contrast, an opposite trend was apparent during the later stages of maturation (Supplementary Fig. S4). To uncover the underlying drivers of the reverse hourglass pattern, we explored the TAI contributions of individual phylostratum. The two oldest PS (PS1 and PS2) contributed the highest with the TAI during embryogenesis and germination (Fig. 1C and Supplementary Fig. S5). However, there was a noticeable decline in their contribution during the transition from embryogenesis (bent cotyledon stage) to maturation (mature green stage) (Supplementary Fig. S6A). In contrast, the contribution of several younger PS (e.g. PS6 [Spermatophyta], PS9 [Pentapetalae], PS12 [Brassicales], and PS13 [Brassicaceae]) was higher during maturation compared to embryogenesis (Fig. 1C). Upon examining the relative expression levels of all PS, we observed a consistent pattern: older PS exhibited higher relative expression during embryogenesis and germination, while younger PS had increased relative expression primarily during maturation (Supplementary Fig. S6A–C). By sorting the genes based on their average expression during the maturation (stages mature green to dry seed), we discovered that several of the top-expressed genes encode for proteins that are well-known for their role in seed filling and desiccation tolerance (Angelovici et al. 2010; Verdier et al. 2013; Leprince et al. 2016). For example, some of the most highly expressed genes from PS9 included seed storage albumins (1–4) and late embryogenesis abundant (LEA) proteins (AT2G21490—dehydrin LEA, AT3G50980—*XERO1*, and AT2G42560—*LEA25*) (Supplementary Table S5). The high expression of these storage protein-encoding genes from PS9 clearly coincides with the initiation of seed-filling during maturation (Baud et al. 2002). Likewise, PS12 (Brassicales) and PS13 (Brassicaceae) also showed an increased contribution during early maturation but reached the highest magnitude at later stages of maturation (18 DAP onwards) (Fig. 1C). For these two PS, the most highly expressed genes also included LEAs and dehydrins, along with several glycine-rich and proline-rich proteins, and hydroxyproline-rich glycoprotein family proteins (Supplementary Table S5). The contributions of PS6 (Spermatophyta) and PS7 (Magnoliopsida) were also noticeable, particularly at later maturation time points, and the highest expressed genes included defensins, LEAs, and lipid transport proteins (Supplementary Table S5).

This insight from individual phylostratum contributions prompted us to delve further into the specific genes and pathways shaping the seed maturation phylotranscriptome landscape. We were specifically interested in identifying genes that drive the high TAI pattern during maturation. To do this, we first recalculated the TAI of the whole seed life cycle by removing either the top 1% (160 genes), 2% (320 genes), 5% (801 genes), 10% (1,602 genes), or 20% (3,203 genes) genes with the highest expression (average TPM) during maturation stages only (Supplementary Table S6). Then, we tested whether removing these gene sets affected the significance of the reverse hourglass (Supplementary Fig. S7). Removing the top 5% genes was sufficient to eliminate the significant reverse hourglass pattern (*p_rev_* = 0.0724), indicating that these genes primarily drive the pattern during the seed life cycle. Comparing these top 5% maturation genes with the top 5% in embryogenesis and germination revealed that the maturation phase had the highest percentage of younger PS genes, although many of these genes still belonged to PS1 and PS2 (Fig. 1D). Interestingly, the expression (mean log10 TPM) of these top 5% maturation genes, especially from younger PS, was relatively more variable and in some cases higher than that of the top 5% genes of embryogenesis and germination (Supplementary Fig. S8).

We next performed gene ontology (GO) enrichment analysis to identify the biological processes that are associated with the top 5% maturation-expressed genes from the different PS. We classified all PS into three groups—(i) “older” (PS1 and PS2, genes conserved among cellular organisms and eukaryotes), (ii) “intermediate” (PS3 to PS8, genes that originated with the green lineage until the origin of core angiosperms), and (iii) “younger” (PS9 to PS16, all genes that originated afterward). As expected, genes from older PS (PS1 and PS2) were specifically highly enriched for processes related to basic cellular functions such as translation, respiration, autophagy, and response to oxidative stress, as well as some specialized functions such as photosynthesis, defense, and lignin biosynthesis (Fig. 1E). Genes from the older PS group were also involved in embryo development, suggesting an ancestral origin of embryo development genes that are highly expressed during maturation. We also found that the older PS group has a significantly higher number of annotated genes compared to the intermediate (P3-P8) and younger (P9-P16) PS groups (Supplementary Fig. S9). Despite this GO annotation bias, we found that seed maturation-related processes such as oil body biogenesis, lipid storage and transport, and seed dormancy were exclusively enriched in genes from the intermediate PS group (PS3-PS8) and processes associated with mucilage biosynthesis and seed coat development were exclusively enriched in genes from the younger PS group (PS9–PS16) (Fig. 1E). We also found that two seed-specific processes, seed germination and seed maturation, were enriched in both older and intermediate PS. Strikingly, we found that two processes of notable importance for seed maturation and desiccation, response to abscisic acid (ABA) and response to water deprivation, were highly enriched in genes from all PS groups.

Together, our findings show that a higher proportion of younger PS genes with high expression are found during maturation compared to embryogenesis and germination. This highlights their key role in driving the reverse hourglass of the seed life cycle and the diverse origins of maturation-enriched processes.

### The reverse hourglass of seed maturation is conserved across multiple plant species

**Figure 2.**
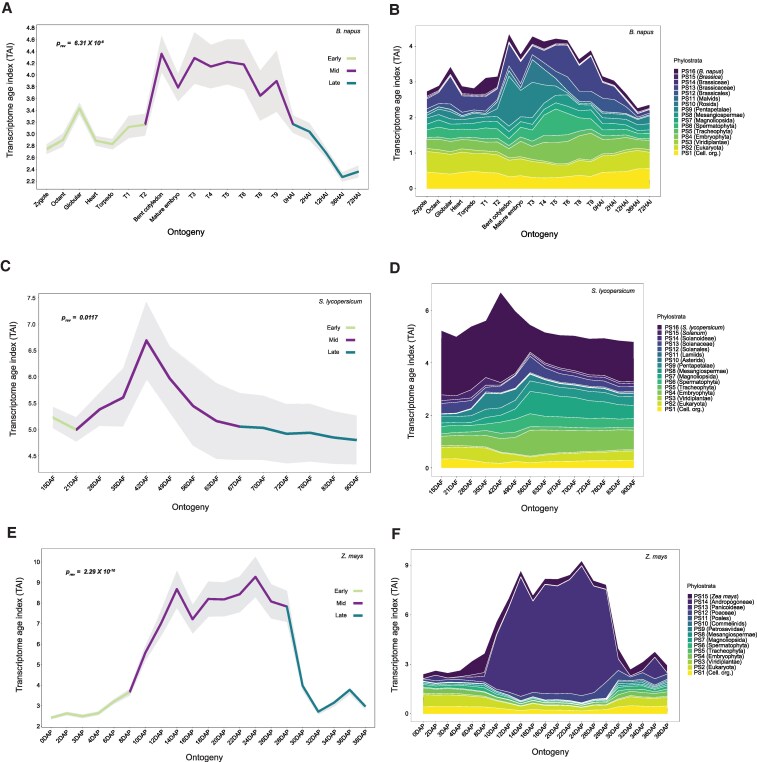
TAI profile during phases of the seed life cycle in three angiosperm species. **A)** TAI profile during the entire seed life cycle in *B. napus*. The colored regions in the mean TAI line indicate stages that were considered part of early, mid, and late development, respectively, while performing the reverse hourglass test. The *P*-value indicates the significance of the test. T1–T9 (thermal time interval) indicates when seeds were harvested during maturation (Bianchetti et al. 2021). HAI indicates hours after imbibition. **B)** Contribution of individual phylostratum to the overall TAI profile in *B. napus*. **C)** TAI profile during parts of the seed life cycle in *S. lycopersicum*. Earlier stages (15 & 21 days after fertilization (DAF) represent the end of embryogenesis, and 28 DAF marks the initiation of seed filling, i.e. maturation (Bizouerne et al. 2021). **D)** Contribution of individual phylostratum to the overall TAI profile in *S. lycopersicum*. **E)** TAI profile during parts of the seed life cycle in *Z. mays*. **F)** Contribution of individual phylostratum to the overall TAI profile in *Z. mays*.

To examine whether high TAI values during seed maturation and the reverse hourglass pattern of the seed life cycle is conserved across angiosperms, we investigated the transcriptome of phases of the seed life cycle of three crop species: the dicots brassica *(Brassica napus,* Brassicacea*)* and tomato (*Solanum lycopersicum*, Solanaceae), and the monocot maize (*Zea mays*, Poaceae). We combined phylostratigraphy of *B.napus* (Supplementary Fig. S10 and Supplementary Table S7) with RNA-seq data covering embryogenesis, maturation, and germination (Supplementary Table S1) (Boter et al. 2019; Bianchetti et al. 2021; Gao et al. 2022) to construct the TAI profile. Similar to Arabidopsis, the *B. napus* maturation time points (mature green until thermal time interval 9, see Methods) exhibited higher TAI compared to stages during early and late development (Fig. 2A). The overall TAI profile also followed a significant reverse hourglass (*p_rev_* = 6.31×10^−6^). We also found that most of the PS with a high TAI contribution during Arabidopsis seed maturation also had higher TAI values in *B. napus* (e.g. PS6, PS9, PS12, and PS13) (Fig. 2B and Supplementary Fig. S11).

For tomato, we calculated the TAI using the gene ages (Supplementary Fig. S12 and Supplementary Table S7) and an RNA-seq dataset that spanned late embryogenesis (15 and 21 days after flowering, DAF) until the end of maturation (90 DAF) (Bizouerne et al. 2021). Despite the absence of early embryogenesis and germination time points, the overall TAI profile also followed a significant reverse hourglass pattern (*p_rev_* = 0.0117) (Fig. 2C). Similar to our findings in Arabidopsis, analysis of individual phylostratum contributions revealed a high TAI from younger PS during tomato maturation (28 DAF onwards) (Fig. 2D and Supplementary Fig. S13A–C). A striking feature was the high contribution of the youngest phylostratum (PS16) during the entire maturation phase; however, removing PS16 genes did not abolish the reverse hourglass pattern (*p_rev_* = 0.00595) (Supplementary Fig. S14), indicating that PS16 gene expression is not the sole driver of the pattern in tomato.

Considering our finding of a conserved reverse hourglass pattern in three dicots (Arabidopsis, *B. napus*, and tomato), we asked if a similar trend was also conserved in a monocot species. To investigate this, we generated the TAI profile of maize using PS information (Supplementary Fig. S15 and Supplementary Table S7) and an existing RNA-seq dataset that covered both embryogenesis (0 DAP to 10 DAP) and maturation (12 DAP to 38 DAP) (Chen et al. 2014) (Supplementary Table S1). We identified a similar reverse hourglass pattern (Fig. 2E, *p_rev_* = 2.29×10^−10^), with a high TAI profile during maturation (12 DAP until 38 DAP) primarily driven by a single phylostratum, PS13 (Panicoideae) (Fig. 2F and Supplementary Fig. S16A–C). By examining average expression during maturation, we identified several zein-encoding genes from PS13 as the most highly expressed causal genes (Supplementary Tables S8 and S9). Zeins are widely known as the most abundant seed storage proteins in maize (Schmitz et al. 1997; Woo et al. 2001; Flint-Garcia et al. 2009). Although the removal of zein genes from PS13 did not affect the reverse hourglass significance (*p_rev_* = 0.0336), it profoundly reduced the TAI peak during maturation (Supplementary Fig. S17A and B). In contrast to PS13, most of the remaining PS (PS3-PS11) had a negligible contribution to the maturation TAI (Fig. 2F and Supplementary Fig. S17B). This represented a clear deviation from the overall pattern observed for the three dicot species investigated here, where multiple younger PS exhibited higher TAI values throughout seed maturation. On the other hand, the TDI profile showed significance for the reverse hourglass test only in *Z. mays* (*p_rev_* = 0.00371, Supplementary Fig. S18C). The test did not yield a significant *P*-value for *B. napus* and *S. lycopersicum* (Supplementary Fig. S18A and B).

**Figure 3.**
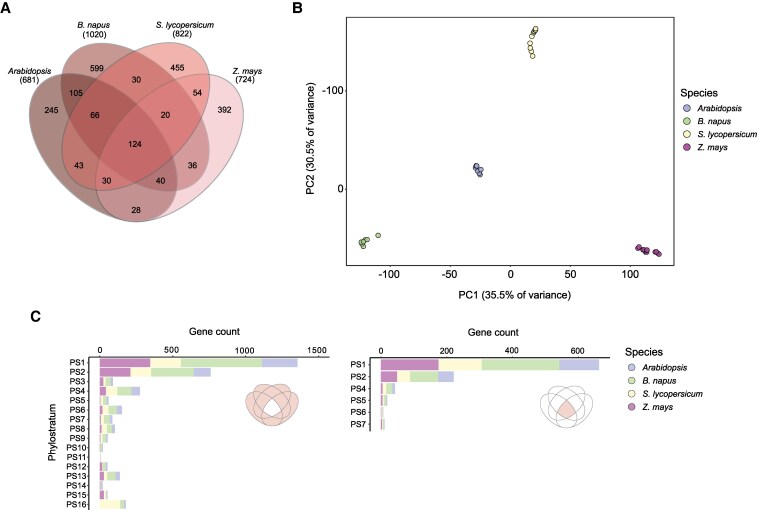
Orthogroup comparison of the top 5% maturation genes in four angiosperm species. **A)** Venn diagram showing the overlap in orthogroups that are represented within the top 5% maturation genes from Arabidopsis, *B. napus*, tomato (*S. lycopersicum*), and maize (*Z. mays*). **B)** PCA showing clustering of maturation time points from all four species based on the top 5% maturation orthogroups shown in the Venn diagram. **C)** Gene counts of orthogroups sorted according to PS that are either conserved (right) or non-conserved (left) in all four species.

To assess the (dis)similarity of the seed maturation transcriptome between these crop species and *Arabidopsis*, we converted the top 5% maturation genes from each species to their respective orthogroups (Supplementary Tables S6 and S9). This led to 681 orthogroups for *Arabidopsis*, 1,020 for *B. napus*, 822 for *S. lycopersicum*, and 724 for *Z. mays*. From these, a total of 124 orthogroups were shared across all four species (Fig. 3A). Plotting the maturation stages of all four species in a PCA based on the median expression (TPM) of the top 5% orthogroups showed a clear distinction among the species with no overlap (Fig. 3B). However, this separation largely disappears when the PCA was performed with only the 124 shared orthogroups (Supplementary Fig. S19). The orthogroups partially shared among the four species (referred to as non-core orthogroups) included genes from nearly all PS in each species, except for PS11 genes in *B. napus* (Malvids) and *Z. mays* (Poales) (Fig. 3C, left). The core maturation orthogroups, which are shared by all four species, consist of genes from only six distinct and predominantly older PS. Most of these genes originated from PS1 and PS2 (Fig. 3C, right) and were enriched for basic cellular processes (using functional annotations of Arabidopsis genes), while genes from younger PS (PS4 to PS7) were enriched for seed-related functions (Supplementary Fig. S20A). On the other hand, genes from the non-core orthogroups were enriched for a range of diverse functions including primary metabolism, responses to stress, and seed development (Supplementary Fig. S20B).

In summary, these findings show that the developmental reverse hourglass pattern during the seed life cycle is conserved across both dicot and monocot species, and the high TAI during seed maturation is mainly driven by high expression of genes from a few relatively younger PS involved in both essential cellular processes and seed-specific functions.

### Embryo and endosperm tissues contribute differentially to the seed maturation TAI

**Figure 4.**
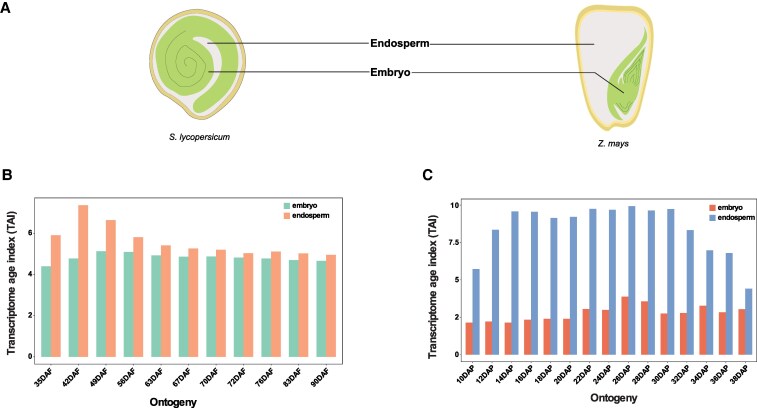
Tissue-specific TAI distribution in *S. lycopersicum* and *Z. mays*. **A)** Schematic representation of *S. lycopersicum* and *Z. mays* seeds showing embryo and endosperm tissues. **B)** TAI of embryo and endosperm tissues during the different stages of *S. lycopersicum* seed development. **C)** TAI of embryo and endosperm tissues during the different stages of *Z. mays* seed development.

To better understand the cause(s) underlying the high TAI profile during seed maturation, we investigated the tissue-specific contribution. This was facilitated by the availability of endosperm and embryo-specific RNA-seq data for a subset of the seed development samples from tomato and maize (Fig. 4A, Supplementary Table S1). In tomato, both tissues had a very similar TAI distribution, except that the endosperm TAI was higher at all-time points (Fig. 4B). The difference in TAI between the two tissues was most pronounced at mid-maturation stages, specifically between 35 and 56 DAF. The higher TAI values of endosperm tissues were most likely due to the contribution of PS6 and PS13 (Supplementary Fig. S21). In maize, the contrast in tissue-specific TAI profiles was clearly discernible, with the endosperm TAI values being magnitudes higher than the embryo (Fig. 4C). The high TAI profile of PS13 observed in the whole maize seed tissue (Fig. 2F) appeared to be almost exclusively due to the endosperm (Supplementary Fig. S22). A likely reason is that the zein genes, which are the dominant genes from PS13 affecting the TAI profile, are expressed particularly in the endosperm (Woo et al. 2001). To test this, we recalculated the endosperm TAI for all PS after removing all genes annotated as zein-encoding (49 genes) from the data (Supplementary Table S8 and Supplementary Fig. S23). As expected, this abolished the high TAI pattern of PS13 in the endosperm, which resulted in a pattern that was more similar to that of the embryo (Supplementary Figs. S22B, S23A, and B).

To discern whether the endosperm-specific contribution observed in maize is species-specific or conserved across monocots, we analyzed a seed development transcriptome dataset from barley (*Hordeum vulgare* L.) (Kovacik et al. 2024). The dataset included RNA-seq samples of whole seeds, embryo, endosperm, and maternal tissues separately (Supplementary Table S1). The dataset covered part of embryogenesis (4 DAP and 8 DAP) and maturation (16 DAP, 22 DAP, and 32 DAP) (Bartels et al. 1988). Similar to the other species, the TAI pattern of barley whole seed transcriptome also increased during the transition from embryogenesis to maturation (Supplementary Fig. S24A and B) (*P*-value for a flatline test, *p_flt_* = 0.024) due to expression of genes from younger PS (Supplementary Fig. S25A). Tissue-specific analysis revealed that similar to maize, the endosperm tissue exhibited higher TAI levels than both embryonic and maternal tissues (Supplementary Fig. S25B). This was due to the near exclusive contributions of PS8, PS12, and PS14 to the barley endosperm TAI pattern (Supplementary Fig. S25B and C). The top 20 genes with the highest expression during these time points (4 DAP to 32 DAP) include, among others, genes encoding for LEA protein, defensins, glutenins, globulin, beta purothionins, alpha-amylases, gamma gliadins, and lipid transfer proteins, which are genes involved in desiccation tolerance and grain filling (Gorjanović 2009; Yang et al. 2023) (Supplementary Table S10).

These findings indicate that the significant role of endosperm-expressed genes in shaping the overall seed TAI pattern is a common feature among monocots. In this context, genes associated with seed desiccation and storage proteins, derived from younger PS, are primarily expressed in the endosperm.

### Functionally specialized genes drive the high TAI patterns of the seed life cycle and pollen development

**Figure 5.**
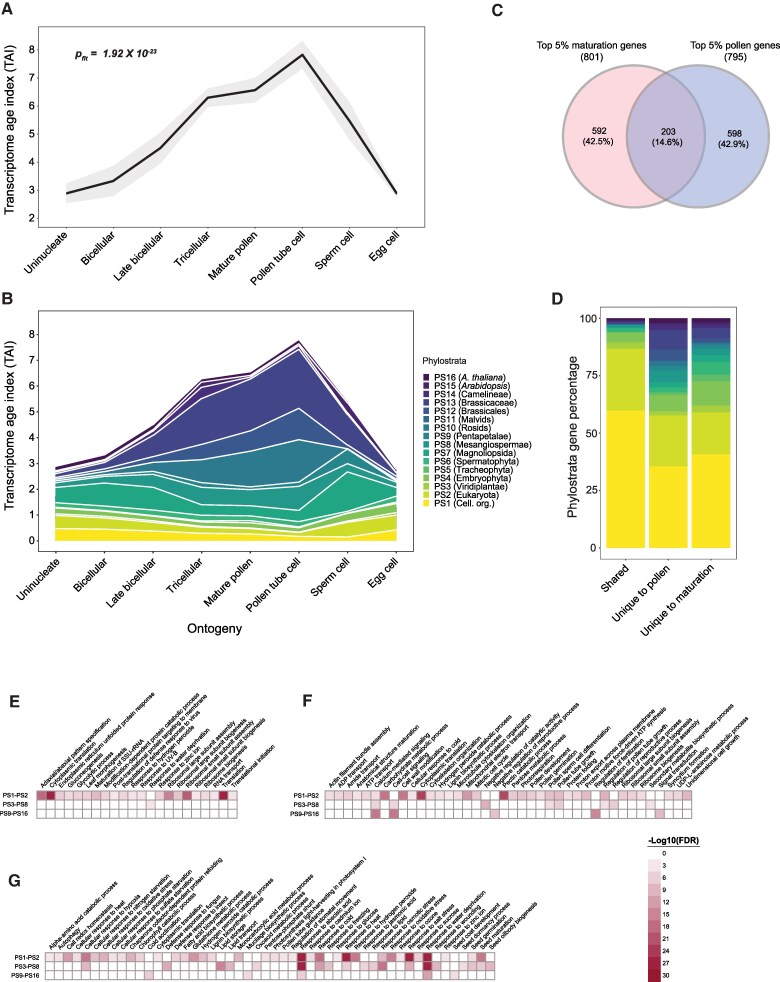
Comparison of the phylotranscriptome of Arabidopsis pollen development and seed maturation. **A)** TAI pattern during Arabidopsis pollen development and egg cell stage. The *P*-value indicates the significance of deviation from a flatline test. Shaded regions around the TAI line indicates the standard deviation (s.d.) calculated from 50,000 permutations. **B)** Contribution of individual phylostratum to the overall pollen TAI profile. **C)** Venn diagram showing the overlap between top 5% genes during seed maturation and pollen development. **D)** Relative percentage of genes from each phylostratum comprising genes that are shared and unique between the seed maturation and pollen development. **E**, **F)** Heatmap showing GO terms that are enriched in genes that are shared, unique to pollen development, and unique to seed maturation, respectively. The color indicates the -log10(FDR) value between a range from 0 to 30.

“Out of the testis” is a widely accepted hypothesis concerning de novo gene evolution that pinpoints that male reproductive tissues serve as the basis for the expression of new genes (Levine et al. 2006; Vinckenbosch et al. 2006; Kaessmann 2010). A multitude of evidence has accumulated over the years in support of this hypothesis for both animal and plant species (termed “out of the pollen” hypothesis in plants) (Betran 2002; Begun et al. 2007; Wu et al. 2014; Cui et al. 2015; Assis 2019; Julca et al. 2021). Given that a high TAI profile has also been reported for pollen development (Cui et al. 2015), we examined the overlap between young PS genes expressed during pollen development and seed maturation. To achieve this, we reanalyzed the RNA-seq data from Arabidopsis (ecotype Col-0) regarding pollen development from Cui et al. (2015) to calculate the TAI values for each phylostratum. We took along the egg cell stage from the same pollen transcriptome data as a reference for the female gamete. The TAI values showed a progressive increase during stages of pollen development and reached a maximum value in the pollen tube cells, while the egg cell TAI value was strikingly lower (Fig. 5A). The overall pattern deviated significantly from a flat line (*p_flt_* = 4.38 × 10^−14^), indicating the presence of an evolutionary signature and, as expected, several younger PS highly contributed to the TAI during pollen development (Fig. 5B and Supplementary Fig. S26) (Cui et al. 2015; Gossmann et al. 2016; Julca et al. 2021).

We then questioned whether similar genes underlay the high TAI profile during pollen development and seed maturation in Arabidopsis. Similar to what we observed for seed life cycle TAI (Supplementary Fig. S7C), removing the top 5% expressed genes from the pollen development dataset (uninucleate until sperm cells, 795 genes) (Supplementary Table S11) largely decreased the high TAI profile (Supplementary Fig. S27). Comparison of the top 5% expressed genes during pollen development and seed maturation (Supplementary Table S6) showed an overlap of 203 genes, with 592 and 598 genes unique to pollen development and seed maturation, respectively (Fig. 5C). Over 80% of the shared genes belonged to older PS (PS1 and PS2) (Fig. 5D). In contrast, a higher percentage of younger PS genes was observed among the unique genes for both pollen development and seed maturation (Fig. 5D). GO enrichment analysis further confirmed that shared genes between pollen development and seed maturation were primarily associated with basic cellular processes such as translation, mRNA maturation, and energy metabolism (Fig. 5E), while highly expressed genes specific to each structure were enriched for functions unique to pollen or seed development (Fig. 5E–G, respectively). These findings show that, while pollen and seed development share a similar reverse hourglass, the genes driving these patterns are functionally specialized for each structure.

### The reverse hourglass pattern is a robust feature of the seed life cycle

To finally validate the robustness of the reverse hourglass and rule out the possibility that the pattern across species was not an artifact of data merging, we performed phylotranscriptome analysis of a recently released RNA-seq data of soybean (*Glycine max*) seed development generated under uniform conditions (Chen et al. 2024; Supplementary Table S1). The data included all three phases in the same RNA-seq. Our analysis revealed a significant reverse hourglass pattern (*p_rev_* = 1.66×10^−08^), with the maturation phase showing higher TAI than the adjacent embryogenesis and germination phases (Supplementary Fig. S28A). Similar to our observation in Arabidopsis, *B. napus*, and tomato, the high TAI during soybean seed maturation was supported by the contribution of several younger PS (Supplementary Fig. S28B). Together, these results demonstrate that the reverse hourglass pattern during the seed life cycle is robust and conserved across both model and crop species, emphasizing the central role of seed maturation.

## Discussion

**Figure 6.**
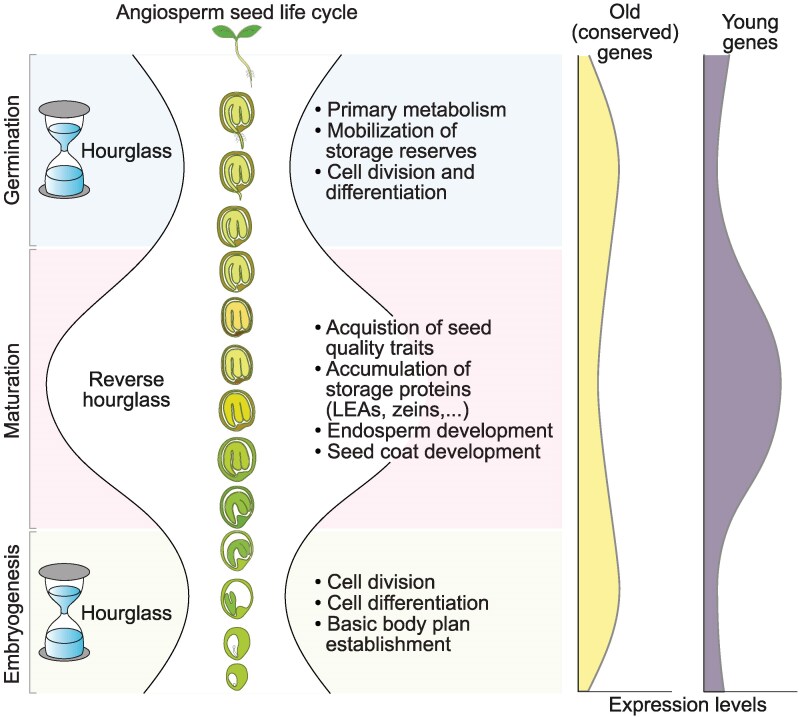
Developmental reverse hourglass model of angiosperm seed life cycle. Schematic presentation of the reverse hourglass model of the seed life cycle indicating the mid-development phase (maturation) characterized by expression of younger genes involved in seed-specific traits, while during the flanking phases (embryogenesis and germination) expression of older genes involved in basic cellular processes is more dominant.

In plants, an hourglass-like development has been reported for embryonic (embryogenesis) and also for post-embryonic (germination) development using the model plant Arabidopsis (Quint et al. 2012; Drost et al. 2016). The general hourglass model of evolution suggests that early and late stages of development are subject to positive selection, leading to molecular and phenotypic divergence. In this work, we implemented similar principles of molecular evolution on the entire seed life cycle in Arabidopsis by treating embryogenesis, maturation, and germination as the early, mid, and late developmental stages, respectively. Intriguingly, unlike embryo development, the entire seed life cycle of Arabidopsis mirrored a reverse hourglass pattern with signatures of a younger transcriptome and higher molecular divergence during the maturation phase. This could indicate that seed maturation experiences higher positive selection compared to embryogenesis and germination, thereby leading to higher genetic and transcriptome divergence across species. It can be argued that the germination phase plays a much more decisive role in the fitness and survival of the seedling and, thus, is a more suitable phase for experimenting with new genes. However, it is well known that processes that occur during maturation, such as the accumulation of storage transcripts and proteins, nutrient reserves, dormancy levels, extent of longevity, etc., directly influence the timing and success of seed germination (Rajjou et al. 2004; Nakabayashi et al. 2005; Fait et al. 2006; Holdsworth et al. 2008). Moreover, a recent study in Arabidopsis showed that a de novo gene, AT1G03106 (SWK) played a role in seed germination under drought stress (Jin et al. 2025). Based on the RNA-seq data used in the present work, this gene shows expression during seed maturation (bent cotyledon stage to mature dry stage) (Supplementary Data S1). This observation further reinforces the claim that maturation-expressed young genes can directly influence adaptive traits crucial during seed germination. According to the reverse hourglass pattern, the early and late stages of the seed life cycle (embryogenesis and germination, respectively) may undergo purifying selection, while seed maturation experiences positive selection. Such positive selection during maturation could harbor ecological and adaptive consequences for both seed maturation and germination. Thereby, testing if newly evolved genes during maturation can lead to phenotypic consequences during both maturation and germination, could allow the fixation of these young genes. Taken together, our findings identify a reverse hourglass-like transcriptome pattern during the angiosperm seed life cycle where more conserved genes are expressed in early (embryogenesis) and late (germination) phases, while the mid-phase (maturation) features a more diverse transcriptome with younger genes (Fig. 6).

We analyzed maturation datasets of multiple angiosperm species spanning both dicot and monocot lineages. Our analyses show that the hourglass pattern across species is robust to data transformations (Supplementary Fig. S2) and not an artifact of data merging (Supplementary Fig. S28A). In all cases, we found that the TAI value increases post-embryogenesis and drops again either during late maturation (maize and tomato) or during germination (Arabidopsis, *B. napus*, and soybean). We then investigated how the phylotranscriptome patterns during the maturation phase behave across species, specifically whether the young maturation transcriptome is conserved among both closely and distantly related species. We found that when considering only the top 5% most highly expressed genes, the maturation transcriptome of four species was highly distinct (Fig. 3B). Intriguingly, this difference was evident for species from the same family (i.e. Arabidopsis and *B. napus*). This is most likely due to the differences in the orthogroups that comprise the top maturation transcriptome among the four species. Moreover, this difference largely disappears when the maturation transcriptome is compared based on the top orthogroups conserved among all four species (Supplementary Fig. S19). Most of the genes from these conserved orthogroups represent genes from older PS, while the genes of non-conserved orthogroups have a diverse PS distribution, with many genes from young PS. This suggests that the maturation transcriptome exhibits considerable variation across species, with younger PS genes playing a crucial role in driving this variability.

We observed that, in dicots, the high TAI during maturation was due to the cumulative contribution of genes from several younger PS (Figs. 1C, 2B, and D), while in monocots (maize and barley), a few young PS played a deterministic role in the overall phylotranscriptome pattern (Fig. 2F and Supplementary Fig. S17). Many of these young PS genes from maize and barley encode proteins related to seed storage or defense (Supplementary Tables S9 and S10). The underlying cause of this difference between dicots and monocots remains inconclusive. One possibility could be the long history of domestication of cereal crops with respect to seed-related traits (Wright et al. 2005; Purugganan and Fuller 2009; Meyer and Purugganan 2013). Domestication may have led to the selection and amplified expression of certain young PS genes that may have contributed to seed size or nutrient content. Future studies comparing the phylotranscriptome during seed maturation of domesticated cereals with their wild relatives could shed light on this. Another distinct pattern of monocots was the selective contribution of endosperm-expressed young genes to the overall high TAI pattern (Supplementary Figs. S22 and S23). This can be explained by the persistent nature of the endosperm in monocots (Olsen 2004; Sreenivasulu et al. 2010; Sreenivasulu and Wobus 2013). On the contrary, in dicots, after cellularization, the embryo gradually absorbs the endosperm during maturation (Berger 1999; Berger et al. 2006; Sreenivasulu and Wobus 2013). As a result, the endosperm is the major compartment for protein storage and nutrient reserves in monocots, while in dicots, they are mainly localized in the cotyledons (Sreenivasulu and Wobus 2013). The increased expression of specific storage protein-associated genes from younger PS primarily drives the reverse hourglass pattern observed in the monocots analyzed in this study. Therefore, the variation in TAIs during seed maturation likely explains lineage-specific endosperm development, physiology, and molecular characteristics in monocots and dicots. It is important to note that the reverse hourglass pattern may be sensitive to log transformation, particularly because only a small set of young genes show extremely high expression during maturation. This effect is especially pronounced in domesticated species such as maize, where just a few zein genes dominate the pattern (Supplementary Table S8). Compressing their expression values with log transformation can therefore mask the distinct contribution of these and other young, maturation-specific genes to the phylotranscriptome.

Exploring the biological processes underlying the Arabidopsis maturation phylotranscriptome showed an enrichment for seed-related and stress-related functions (Fig. 1E). In most angiosperm seeds (termed “orthodox” seeds), a desiccation phase occurs at later stages of maturation, and thus, osmotic stress is an inherent feature of seed development (Ooms et al. 1993; Dekkers et al. 2015; Leprince et al. 2016). Our findings showed that biological processes related to ABA response and water deprivation were enriched in the top 5% of maturation-expressed genes. This enrichment was due to maturation-expressed genes spanning all three PS age groups. This indicated that both old and young genes contributed toward the evolution of osmotic stress tolerance during seed maturation. Among the most well-known examples of desiccation-associated genes highly expressed during seed maturation, we found several *LEA*s (Hundertmark and Hincha 2008; Artur et al. 2019) from a broad range of PS among the most highly expressed genes in both Arabidopsis and maize maturation (Supplementary Table S12). Despite their undeniable association with desiccation stress, several questions still remain about the relationship between the evolution of LEAs and their structural diversity and molecular functions in response to cellular water loss (Hernández-Sánchez et al. 2022). Our findings show that the combined high expression of *LEA*s from diverse origins underlies seed maturation and might be required for desiccation tolerance acquisition in seeds. Our finding that desiccation and osmotic stress-related processes are enriched in young PS genes during Arabidopsis maturation supports the well-established link between young genes and stress responses (Donoghue et al. 2011). This association has been documented in various species. In sugarcane (*Saccharum* spp.), lineage-specific orphan genes are enriched for stress and defense responses, especially under cold and osmotic stress (Cardoso-Silva et al. 2022). In rice, Poaceae, and Oryzeae-specific genes are also enriched for stress and defense functions (Stein et al. 2018). Similar patterns have also been reported in more distantly related organisms. In yeasts, de novo genes, including those from non-coding regions, are associated with specific stress-related functions, and younger genes are more likely to be expressed under stress, thereby promoting the functionalization of newly emerged genes (Carvunis et al. 2012; Doughty et al. 2020; Blevins et al. 2021). In *Daphnia*, lineage-specific genes are enriched in the transcriptome from ecologically challenging conditions (Colbourne et al. 2011).

*De novo* genes are well known to be expressed in male reproductive tissues (Betran 2002; Levine et al. 2006; Kaessmann 2010). Even within angiosperms, multiple studies have shown that younger genes (including those of de novo origin) are more likely to be expressed in the male gametes (Williams 2008; Wu et al. 2014; Cui et al. 2015; Gossmann et al. 2016). By comparing the phylotranscriptomic patterns of both Arabidopsis pollen development and seed maturation, we observed a higher TAI owing to high expression of multiple young PS genes. A cross-comparison of the top-expressed genes in both structures showed a partial overlap, mostly consisting of genes from older PS involved in basic cellular functions. Despite the similar reliance on young genes, both stages of development seem to express a unique set of genes that are functionally specialized to the respective developmental stage. Due to the relevance of pollens and seeds in natural selection and in the evolution of angiosperms, these new genes may get fixed by gaining specialized functions related to either of the two developmental stages (Stanton et al. 1986; Moles et al. 2005; Williams 2008; Sims 2012; Coen and Magnani 2018).

Based on our findings, we propose the “out of the seed” hypothesis for the evolution of novel genes during seed maturation. This is similar to the “out of the male” hypothesis widely known in both plants and animals (Begun et al. 2007; Cui et al. 2015; Gossmann et al. 2016; Assis 2019). Our hypothesis highlights how seed maturation can serve as a landscape that offers opportunities to test young genes and facilitate functional specialization.

## Materials and methods

### Transcriptome datasets and RNA-seq analysis

Transcriptome datasets covering the three phases of the seed life cycle (embryogenesis, maturation, and germination) were obtained from publicly available RNA-seq libraries from previous studies (Supplementary Table S1). For Arabidopsis and *B. napus*, samples until the bent cotyledon stage were considered part of embryogenesis (Baud et al. 2002; Bianchetti et al. 2024) and subsequent time points until the dry seed stage were considered part of maturation. For *B. napus*, the RNA-seq time points are expressed as thermal time (T1–T6 and T8–T9) where each time point represents growing degree days from the start of flowering at a base temperature of 0 °C (detailed in Bianchetti et al. 2021). T1–T2 correspond to the transition from torpedo to bent cotyledon stages, T2–T6 to seed filling, and T6–T9 represents the final stages of seed maturation, i.e. late maturation (Bianchetti et al. 2021). Based on the onset of seed filling and maturation traits, the tomato RNA-seq dataset was divided into two parts: 15 DAF to 21 DAF indicating the last stages of embryogenesis, and 28 DAF onwards corresponding to maturation (Bizouerne et al. 2021). The maize transcriptome was grouped into embryogenesis (0 days after pollination [DAP] to 10 DAP) and maturation (12 DAP to 38 DAP) based on the expression of zein genes which were considered as indicators of seed filling (Chen et al. 2014). As for barley, time points 4 DAP and 8 DAP were considered part of embryogenesis while 16 DAP to 32 DAP were considered as part of maturation based on initiation of seed filling (Bartels et al. 1988). The soybean data generated by Chen et al. (2024) covered all three phases of the seed life cycle with heart—cotyledon stages representing embryogenesis, early-maturation—dry seed stages covering maturation, and imbibition to seedling stages covering germination.

Fastq files were downloaded from the National Center for Biotechnology Information (NCBI) using the Sequence Read Archive Toolkit (v3.0.3) and mapped to their respective genomes (indicated in Supplementary Table S1) using the nf-core (Di Tommaso et al. 2017; Ewels et al. 2020) rnaseq pipeline (v3.9). The pipeline uses bedtools (v2.30.0) (Quinlan and Hall 2010), bioconductor-summarizedexperiment (v1.20.0) (Morgan et al. 2022), bioconductor-tximeta (v1.8.0) (Love et al. 2020), fastqc (v0.11.9), gffread (v0.12.1) (Pertea and Pertea 2020), picard (v2.27.4), star (v2.7.10a) (Dobin et al. 2013), salmon (v1.5.2) (Patro et al. 2017), stringtie (v2.2.1) (Pertea et al. 2015), Trimg,alore (v0.6.7), and ucsc (v377). The resulting gene TPM values were used for all downstream analysis.

The Arabidopsis embryogenesis transcriptome dataset (Hofmann et al. 2019) comprised of RNA extracted from embryos only (Fig. 1B, pre-globular—mature green). Whereas, the two other datasets (Artur et al. 2024; Bai et al. 2025) were derived from whole seed tissues (Fig. 1B, 12DAP—germination 72 h) (Supplementary Table S1). The *B. napus* embryogenesis transcriptome dataset comprised of multiple tissues—embryo and seed coat. For these developmental stages (zygote—mature embryo), we used the average TPM across tissues and replicates of the same stage (Supplementary Table S1). The rest of the stages (T1–72HAI) were derived from whole seed tissue. The tomato RNA-seq dataset was generated from embryo, endosperm, and seed coat tissues separately (Supplementary Table S1). TPM counts from all three tissues and their replicates were averaged to retrieve the mean TPM for each developmental stage. The mean TPM values were used to calculate the overall TAI (Fig. 2C) (see below). For generating the tissue specific TAI profiles, only the TPM values of that specific tissue were used for each developmental stage. The maize transcriptome data was also comprised of multiple tissues specifically embryo, endosperm, and whole seed tissue. The replicate averaged TPM values from only the whole seed tissue libraries were used to generate the overall TAI profile (Fig. 2E) while the tissue specific TPM counts were used to generate the tissue specific TAI profiles. The barley TAI profiles were generated in a similar fashion. In the soybean RNA-seq data, sequencing was performed on RNA extracted from embryonic tissue only for heart and cotyledon stages. As for the stages from early-maturation to imbibition stage, read files were generated separately for embryonic axis and cotyledon tissues. For germination and seedling stages, RNA-seq was performed on cotyledon, shoot, and root tissues. For all stages, we averaged the TPM counts over different tissue types to obtain the mean expression at each stage for the whole embryo or seedling.

### Phylostratigraphy

The phylostratigraphy of all plant species was constructed using GenEra (v1.4.0) (Barrera-Redondo et al. 2023) with default parameters. All protein sequences of a given target species were queried against a custom protein database comprising constructed using NCBI refseq genomes of 4,940 species (Supplementary Table S2). The genome data were downloaded using the bash script genome_updater.sh (v0.6.3). The blastp search within the GenEra pipeline was performed using DIAMOND (v 2.1.8) (Buchfink et al. 2021) blastp in more-sensitive mode with an e-value threshold of 10^−5^. The custom database comprised proteomes of 175 plants, 582 fungi, 391 vertebrates, 384 invertebrates, 96 protozoa, 1,055 archaea, and 2,257 bacteria (Supplementary Table S2). Blastp hits were considered as homologs of the query protein and were used to determine gene family founder events and the relative age of a gene.

### TAI calculation

TAI of RNA-seq samples were calculated as described previously (Domazet-Lošo and Tautz 2010) using the myTAI (v2.0.0) (Drost et al. 2015) package in R (v4.4.1). TAI values were calculated as the weighted mean of phylostratum rank (*ps_i_*) of a given gene *i* by the expression level (*e_is_*) in the transcriptome of an RNA-seq sample *s*,


$$TAI_{s}=\frac{\sum_{\begin{array}{c} i=1 \end{array}\;}^{n}ps_{i}\times e_{is}}{\sum_{\begin{array}{c} i=1 \end{array}\;}^{n}e_{is}}$$

Where *n* represents the total number of genes present in the RNA-seq sample *s*. A high TAI value indicates a younger transcriptome age, whereas a lower TAI corresponds to a more ancient transcriptome.

The statistical significance of the phylotranscriptome profiles was evaluated using non-parametric permutation tests such as the flat line test, reductive hourglass test, and reverse hourglass test. These tests were performed using the myTAI package (v2.0.0) (Drost et al. 2018). A flat line tests whether the phylotranscriptome profile deviates from a flat line, whereas a reductive hourglass and reverse hourglass test determine whether a phylotranscriptome pattern visually resembles an hourglass shape (high-low-high) or a reverse hourglass shape (low-high-low), respectively. All tests were conducted with 50,000 permutations.

For the reverse hourglass test, developmental stages of each species were partitioned into three groups—early, mid, and late. For *Arabidopsis* and *B. napus*, embryogenesis time points were grouped as early, maturation time points as mid, and germination time points as late (Supplementary Table S1). On the other hand, the tomato RNA-seq dataset comprised of time points from late embryogenesis (15 DAF and 21 DAF) until the end of seed maturation (90 DAF). In this case, we considered 15 DAF to 21 DAF as early, 35 DAF to 70 DAF as mid, and 72 DAF to 90 DAF as late stages. In the case of tomato and maize, late maturation time points were grouped as late stages. Similarly, for maize, stages 0 to 8 DAP were considered early, 10 DAP to 28 DAP as mid, and 30 DAP to 38 DAP as late stages. For soybean, heart-cotyledon, early-maturation—dry seed, and imbibition—seedling stages were considered part of early-, mid-, and late-development, respectively.

The stability of the reverse hourglass pattern across different RNA-seq transformation methods was calculated using the myTAI package. We tested different transformation methods on the Arabidopsis RNA-seq data—squared, square-root transformation, log transformation with a pseudo-count of 1 (log2[count + 1]), and rank transformation (genes were ranked by level of expression at each stage) were performed on TPM values. Additionally, variance-stabilizing transformation was performed on the raw read counts using DESeq2 (Love et al. 2014).

Relative expression and individual phylostratum contribution were also calculated with the myTAI package in R. The data was plotted using ggplot2 (v3.5.0) (Wickham 2009).

### TDI calculation

The ratio of synonymous substitution rate and non-synonymous substitution rate (Ka/Ks) for *A. thaliana* and *A. lyrata* orthologous gene pairs was determined using the R package orthologr (Drost et al. 2015). The following arguments were used for Ka/Ks calculation, ortho_detection = “RBH”, aa_aln_type = “pairwise”, aa_aln_tool = “NW”, codon_aln_tool = “pal2nal”, dnds_est.method = “Comeron”. Gene pairs with a Ka/Ks < 2 were retained. Based on the Ka/Ks values, Arabidopsis genes were binned into ten divergence strata (DS) from DS1 to DS10 (low to high). The TDI was calculated for the same RNA-seq samples by replacing the PS with the DS, as shown below,


$$TDI_{s}=\frac{\sum_{\begin{array}{c} i=1 \end{array}\;}^{n}ds_{i}\times e_{is}}{\sum_{\begin{array}{c} i=1 \end{array}\;}^{n}e_{is}}$$

A high/low TDI value indicates a conserved/divergent transcriptome. Similarly, the Ka/Ks ratio was calculated for *B. napus*, tomato, and maize genes based on ortholog comparison with *B. oleracea*, *S. pimpinellifolium*, and *Z. diploperennis* genomes, respectively. Subsequently, the TDI values were calculated using the Ka/Ks ratio.

### Determining the top maturation-expressed genes

To determine the top maturation-expressed genes with the highest expression, we calculated the mean TPM expression for each gene over maturation time points (Supplementary Table S1). Genes with an average TPM of ≥1 were taken along and sorted based on average expression from high to low. From this list, the top 5% of genes were considered the top maturation-expressed genes.

### GO enrichment analysis

*A. thaliana* GO slim terms were downloaded from TAIR (https://www.arabidopsis.org/). GO terms for *B. napus*, tomato, and maize proteins were derived from eggNOG-mapper (v2) (Huerta-Cepas et al. 2019; Cantalapiedra et al. 2021) using default parameters. GO enrichment was carried out using the R package topGO (v2.54.0) (Alexa and Rahnenfuhrer 2023) using Fisher's exact test. GO terms with a false discovery rate (FDR) of ≤0.001 were retained. The list was sorted based on −log10(FDR) from highest to lowest, and only the top 15 non-redundant GO terms were shown from each PS group.

### Interspecies maturation transcriptome comparison

Protein sequences of all five species—Arabidopsis, *B. napus*, *S. lycopersicum*, *Z. mays*, and *H. vulgare,* were used to determine orthologous gene groups (orthogroups) using Orthofinder (v2.5.5) (Emms and Kelly 2019).

To perform interspecies transcriptome comparisons, we first filtered the transcriptome of each species to retain genes that have a TPM expression of 2 in at least 3 RNA-seq samples. Next, we converted the gene identifiers (IDs) per species to their respective orthogroups and took the median TPM value per orthogroup. A log2 conversion was performed on the median TPM value with a pseudo count of 1. Subsequently, the converted expression values were used to create PCA plots for different subsets of orthogroups—(i) the top 5% maturation orthogroups, i.e. orthogroups that represented the top 5% maturation genes in each species (Fig. 3A and B). (ii) Shared top 5% maturation orthogroups, i.e. orthogroups that were among the top 5% maturation genes in all four species (Supplementary Fig. S19), and (iii) all shared orthogroups among the four species. Only the developmental stages that belonged to the maturation phase of each species were shown in the PCA.

### Accession numbers

The TPM counts that were used to calculate the TAI values, including the phylostrata information, can be found in Supplementary Data Set 1.

## Supplementary Material

koaf266_Supplementary_Data

## Data Availability

All scripts related to the manuscript have been uploaded to https://github.com/asifahmed23/reversehourglass.

## Supplementary data

The following materials are available in the online version of this article.

**Supplementary Figure S1.** Phylotranscriptomic pattern during the Arabidopsis seed life cycle separated into three major developmental phases.

**Supplementary Figure S2.** Effect of different RNA-seq transformation methods on the significance of the reverse hourglass test in Arabidopsis.

**Supplementary Figure S3.** PCA showing clustering of Arabidopsis RNA-seq samples after data transformations.

**Supplementary Figure S4.** Mean relative expression of two PS classes.

**Supplementary Figure S5.** Percentage contribution of each PS.

**Supplementary Figure S6.** Relative expression of individual PS during Arabidopsis seed life cycle.

**Supplementary Figure S7.** TAI profile over Arabidopsis seed life cycle after removing the top maturation genes.

**Supplementary Figure S8.** Mean expression (log10-TPM) of the top 5% Arabidopsis genes with highest average expression.

**Supplementary Figure S9.** Number of GO annotation terms assigned to Arabidopsis genes from different PS groups.

**Supplementary Figure S10.** Phylostratigraphy of *B. napus* genes.

**Supplementary Figure S11.** Relative expression of each PS during *B. napus* seed life cycle.

**Supplementary Figure S12.** Phylostratigraphy of *S. lycopersicum* genes.

**Supplementary Figure S13.** Relative expression of individual PS during part of *S. lycopersicum* seed life cycle.

**Supplementary Figure S14.** TAI profile during *S. lycopersicum* seed maturation excluding genes from PS16.

**Supplementary Figure S15.** Phylostratigraphy of *Z. mays* genes.

**Supplementary Figure S16.** Relative expression of individual PS during *Z. mays* seed life cycle.

**Supplementary Figure S17.** Phylotranscriptomics pattern of *Z. mays* without zein genes.

**Supplementary Figure S18.** TDI pattern during parts of the seed life cycle in three angiosperm species.

**Supplementary Figure S19.** PCA based on the 124 shared top 5% maturation orthogroups.

**Supplementary Figure S20.** Top GO terms (sorted based on -Log_10_[FDR]) enriched in Arabidopsis genes.

**Supplementary Figure S21.** Tissue-specific individual PS contribution to the overall observed TAI profile in *S. lycopersicum*.

**Supplementary Figure S22.** Tissue-specific individual PS contribution to the overall observed TAI profile in *Z. mays*.

**Supplementary Figure S23.** TAI profile of *Z. mays* endosperm tissue without zein genes.

**Supplementary Figure S24.** Phylotranscriptome of parts of *H. vulgare* seed life cycle.

**Supplementary Figure S25.** Contribution of individual PS to the TAI profile of *H. vulgare* during part of the seed life cycle.

**Supplementary Figure S26.** Percentage contribution of each PS (PS1-PS16) to the overall TAI profile during Arabidopsis pollen development and egg cell stage.

**Supplementary Figure S27.** Arabidopsis pollen TAI profile without top 5% pollen expressed genes.

**Supplementary Figure S28.** TAI profile during the entire seed life cycle in soybean (*Glycine max*).

**Supplementary Table S1.** RNA-seq datasets of the different species used in the study along with their sources.

**Supplementary Table S2.** List of genomes used for determining phylostratigraphy of plant species used in the study. All genomes were downloaded from the NCBI refseq database.

**Supplementary Table S3.** Phylostratigraphy of Arabidopsis genes determined using GenEra (v1.4.0).

**Supplementary Table S4.** Ka/Ks ratio of Arabidopsis genes as compared to *A. lyrata*.

**Supplementary Table S5.** Arabidopsis maturation expressed genes sorted based on corresponding PS.

**Supplementary Table S6.** Top 20% Arabidopsis genes with the highest average expression during maturation time points.

**Supplementary Table S7.** Phylostratigraphy of *B. napus* (Bna), maize (*S. lycopersicum,* Sly), maize (*Z. mays,* Zma), and *H. vulgare* (Hvu) determined using GenEra.

**Supplementary Table S8.** List of zein encoding genes in the maize genome (B73).

**Supplementary Table S9.** List of top 5% genes with the highest average expression (TPM) during seed maturation in the three species—*B. napus*, *S. lycopersicum*, and *Z. mays*.

**Supplementary Table S10.**  *H. vulgare* genes expressed during parts of the seed life cycle sorted from high to low based on average expression (TPM).

**Supplementary Table S11.** Top 5% genes with highest average expression (TPM) during pollen development in Arabidopsis.

**Supplementary Table S12.** List of LEA genes expressed during seed maturation in Arabidopsis.

**Supplementary Data S1.** TPM counts of all the RNA-seq samples used to generate the TAI profile for the four species—Arabidopsis, *B. napus*, *S. lycopersicum*, and *Z. mays*. The first column in each sheet indicates the PS of the genes.

## Dive Curated Terms

The following phenotypic, genotypic, and functional terms are of significance to the work described in this paper:

XERO1 Gramene: AT3G50980
XERO1 Araport: AT3G50980

SWK Gramene: AT1G03106
SWK Araport: AT1G03106
