# Supplementary material for: The phylotranscriptomic profile of angiosperm seed development follows a reverse hourglass pattern
Source: Plant Cell. 2025 Nov 12;37(12):koaf266. doi: 10.1093/plcell/koaf266 (PMC12677925; doi:10.1093/plcell/koaf266)
Supplement: koaf266_Supplementary_Data [file koaf266_supplementary_data.zip › Supplementary Data.pdf]

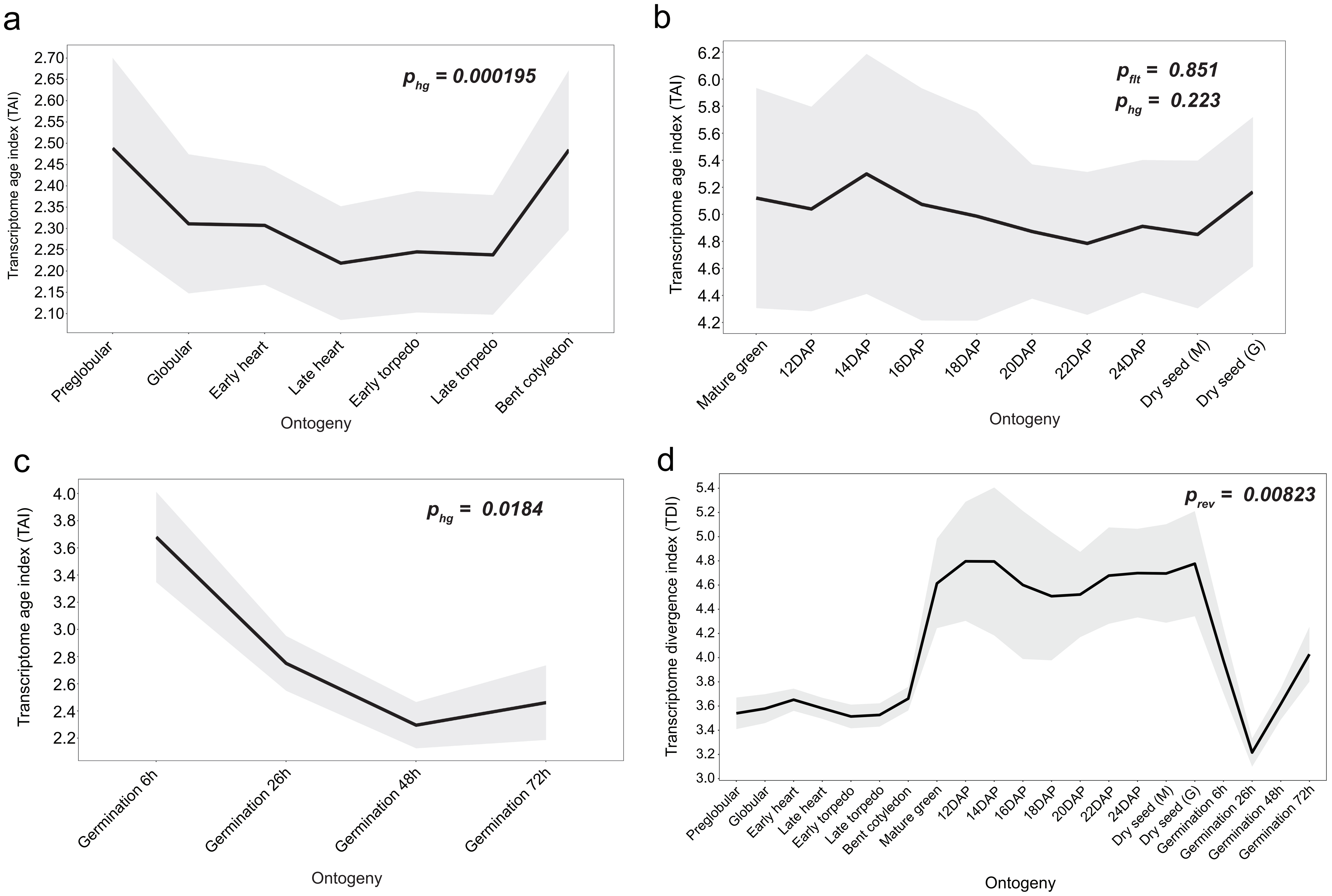

Supplementary Figure S1. Phylotranscriptomic pattern during the Arabidopsis seed life cycle separated into three major developmental phases - **a**, embryogenesis; **b**, maturation; and **c**, germination. P-values shown as p_{hg} and p_{flt} indicate significance for an hourglass and flat line test. **d**, TDI pattern during seed life cycle in Arabidopsis. The overall pattern significantly resembles a reverse hourglass pattern. The grey area indicates strandard deviation calculated based on 50,000 permutations.

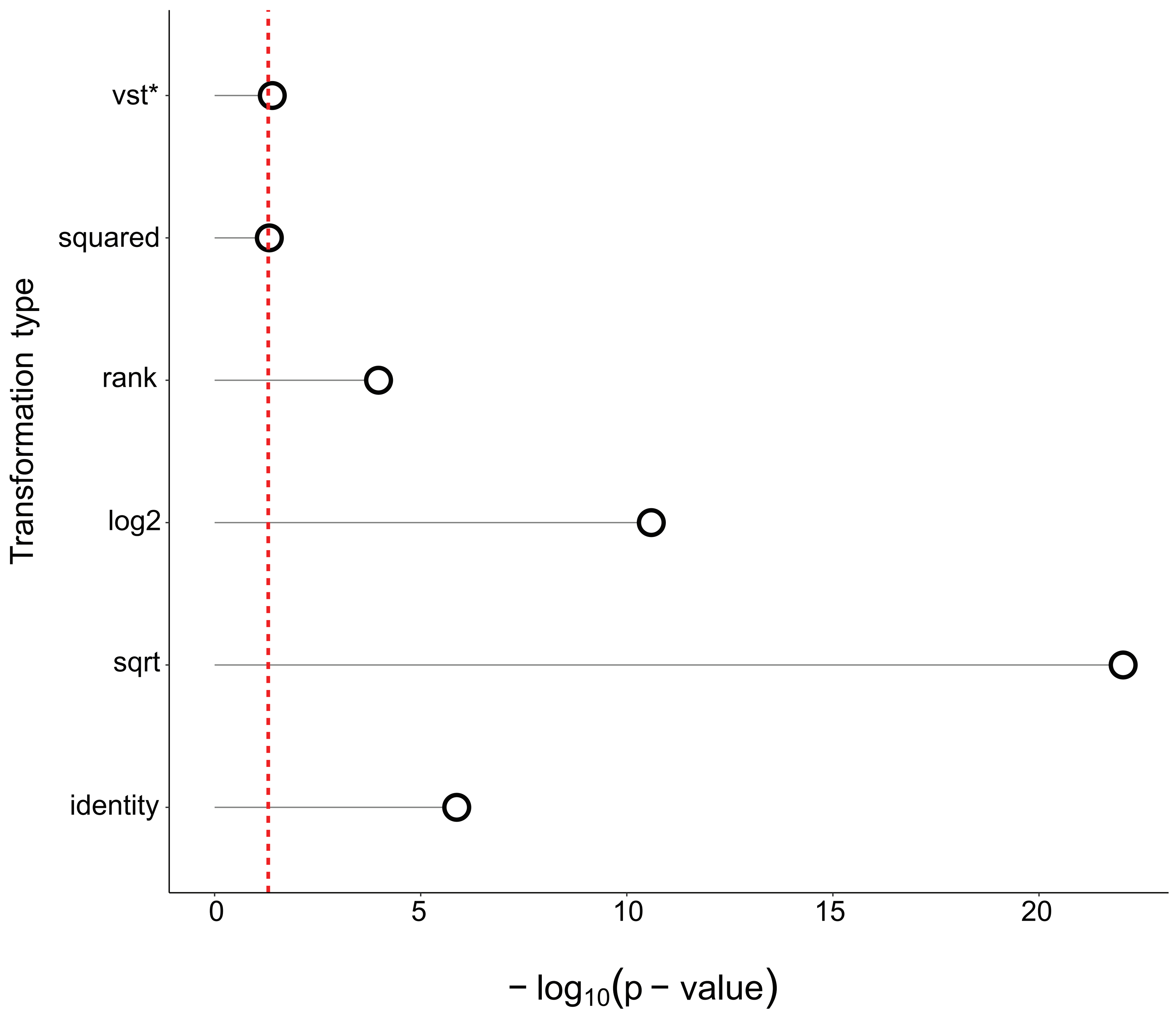

Supplementary Figure S2. Effect of different RNA-seq transformation methods on the significance of the reverse hourglass test in Arabidopsis. The transformations performed were: identity (’none’), square-root (’sqrt’), logarithmic (’log2’), non-parametric rank (’rank’), squared (’squared’), and variance-stabilizing (’vst’). ‘vst’ was performed on the raw read counts. All other data transformations were performed on TPM counts. The circles attached to the lines indicate the p-value for each data transformation. The dashed red line indicates a p-value cutoff of 0.05 on $\log_{10}$ scale and any value higher than the red line represents a p-value lower than 0.05. All tests were performed with 50,000 permutations, and all TAI profiles were significant for a reverse hourglass test. (p-value <0.05). This shows that the pattern observed in Figure 1b is robust to different data transformations.

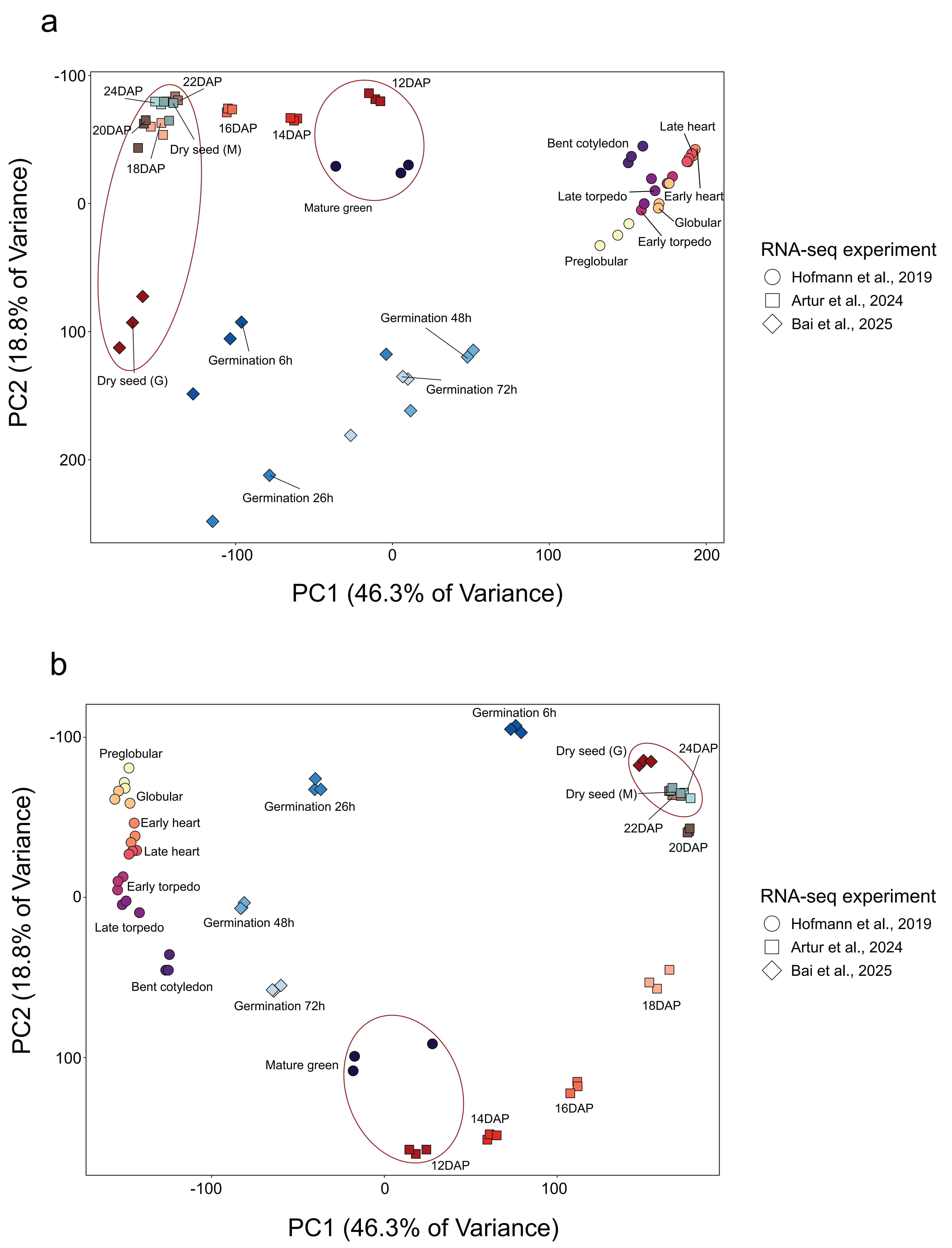

Supplementary Figure S3: PCA showing clustering of Arabidopsis RNA-seq samples after data transformations. **a**. log2 transformation of the TPM counts; **b**, variance stabilizing normalization (VST) using DESeq2. Red ellipses indicate maturation stages from two different sources that cluster together. This indicates that the reverse hourglass pattern in Figure 1b is not an artifact of data merging.

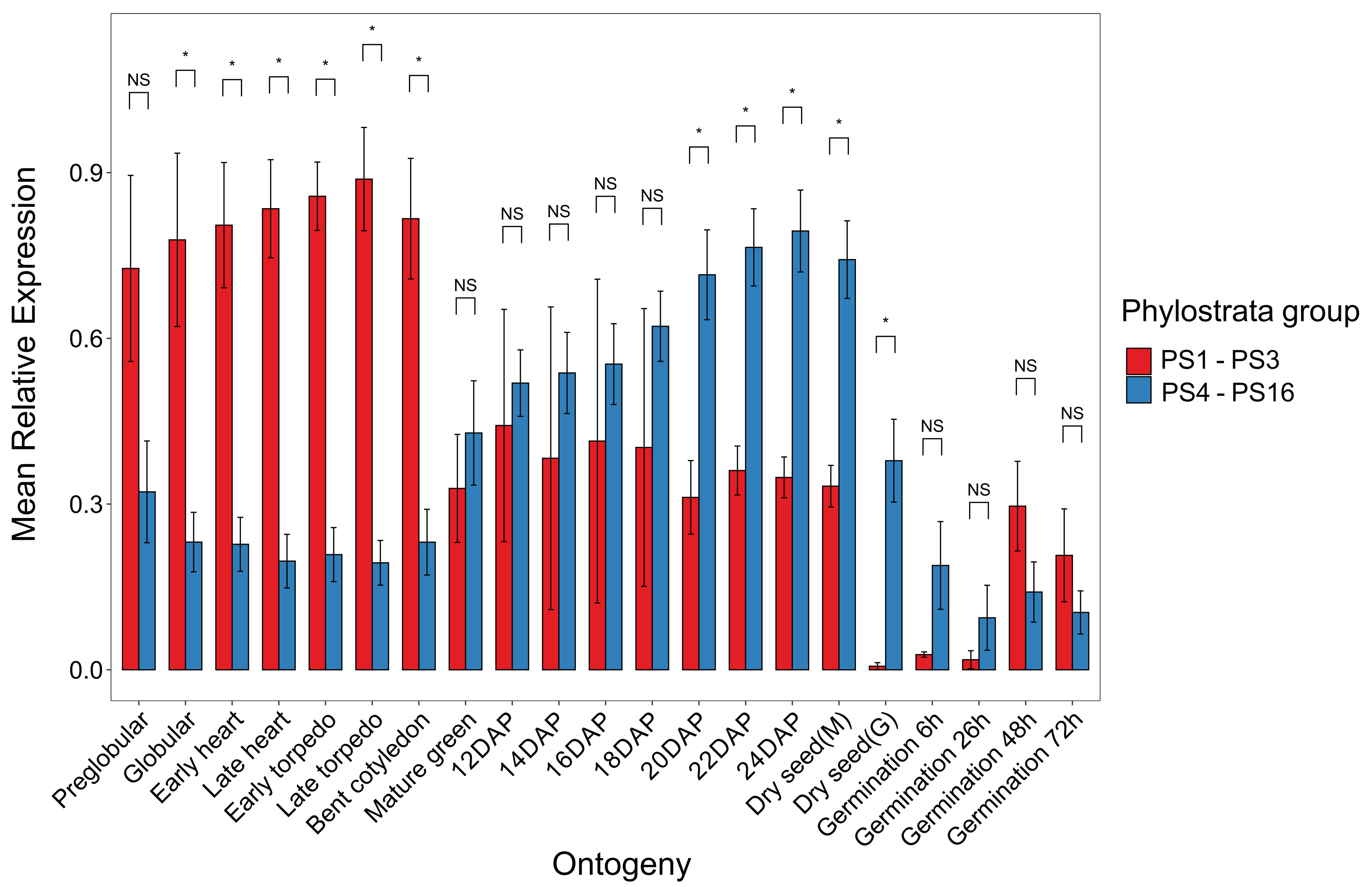

Supplementary Figure S4. Mean relative expression of two phylostrata classes - i. PS1-PS3 (older), and ii. PS4-PS16 (younger) during Arabidopsis seed life cycle. Significance was calculated using the non-parametric Kruskal-Wallis rank sum test between the two phylostrata classes. Significant differences were denoted with an asterix (*). NS indicates that the different is not significant. Errorbars indicate the standard deviation from the mean. This reinforces the findings shown in Figure 1c,d that younger phylostrata genes have a higher contribution during maturation.

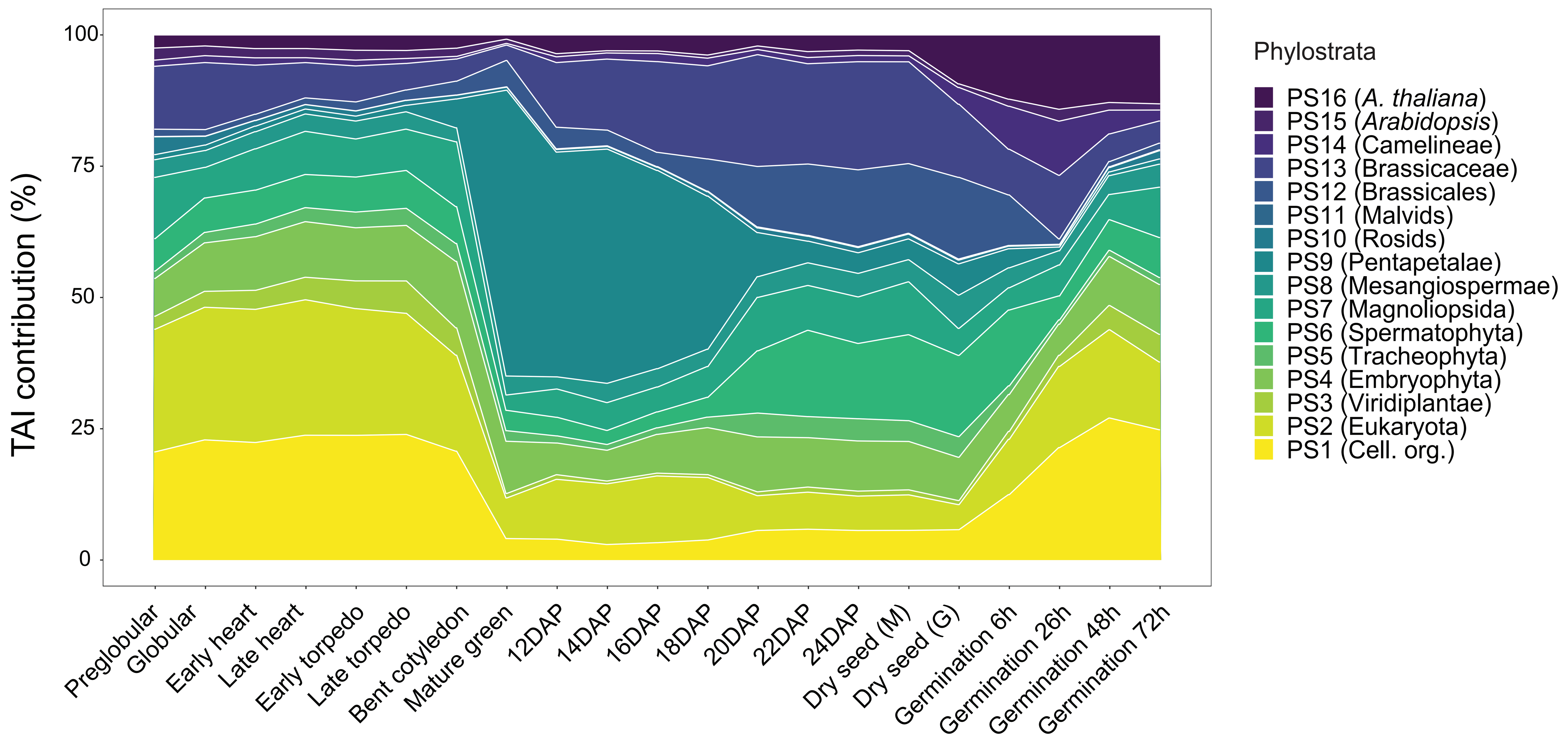

Supplementary Figure S5. Percentage contribution of each phylostrata (PS1-PS16) to the overall TAI profile during Arabidopsis seed life cycle. Similar to Figure 1c, this shows that younger phylostrata comprise the majority of maturation transcriptome TAI.

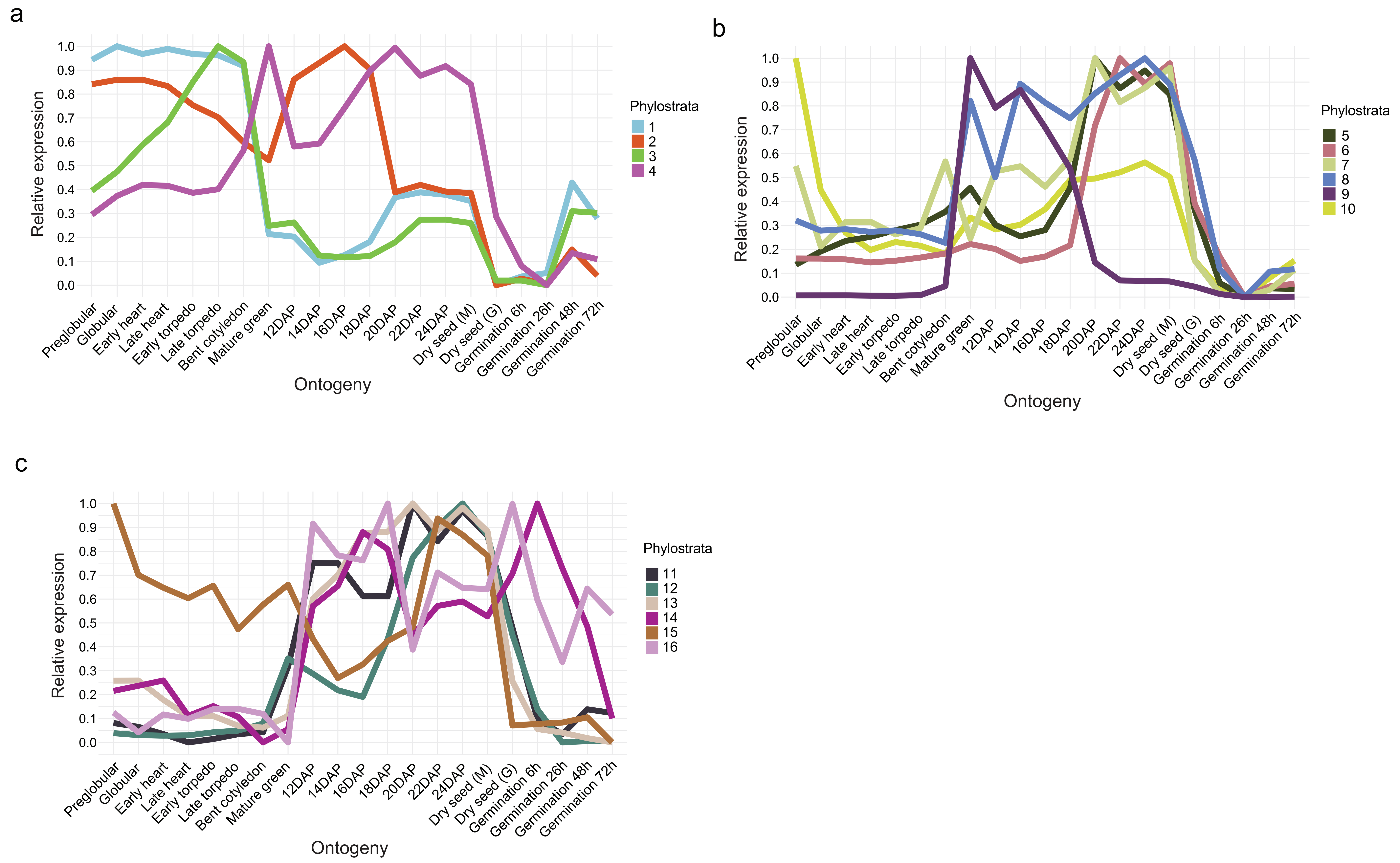

Supplementary Figure S6. Relative expression of individual phylostrata during Arabidopsis seed life cycle. **a**, PS1-PS4; **b**, PS5-PS10; and **c**, PS11-PS16.

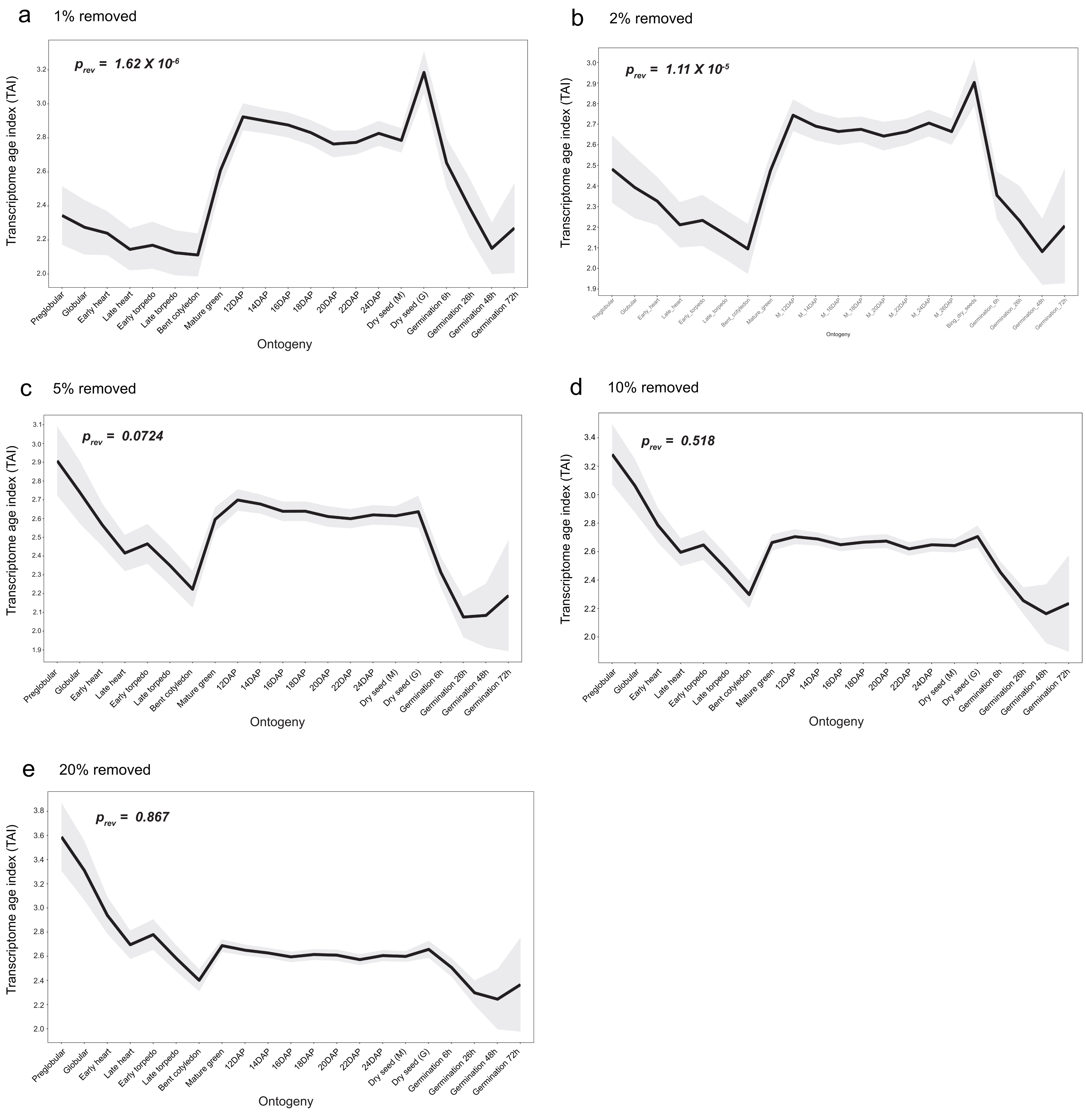

Supplementary Figure S7. TAI profile over Arabidopsis seed life cycle after removing the top **a**, 1%; **b**, 2%; **c**, 5%; **d**, 10%; or **e**, 20% highly expressed genes during seed maturation (Mature green stage to Bing Dry seed). Grey area around the lines indicate standard deviation.

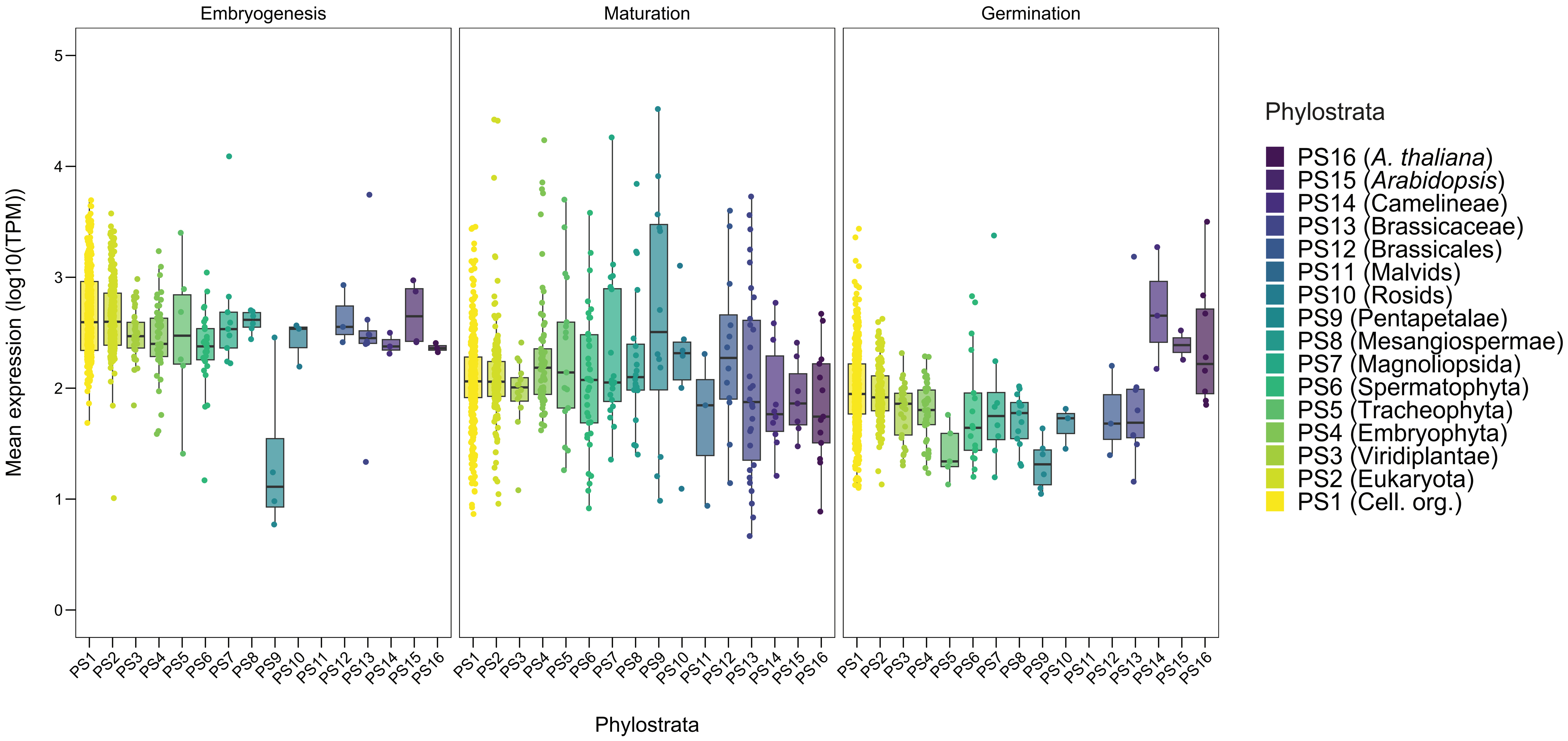

Supplementary Figure S8. Mean expression (log10-TPM) of the top 5% Arabidopsis genes with highest average expression during each of the three phases of the seed life cycle. From left to right – top 5% embryogenesis expressed genes, top 5% maturation expressed genes, and top 5% germination expressed genes. Horizontal line in the middle of the boxes represent the group mean. Upper and lower edges of each box represent the 75th and 25th percentile, respectively. Whiskers represent the variability of the data within each group.

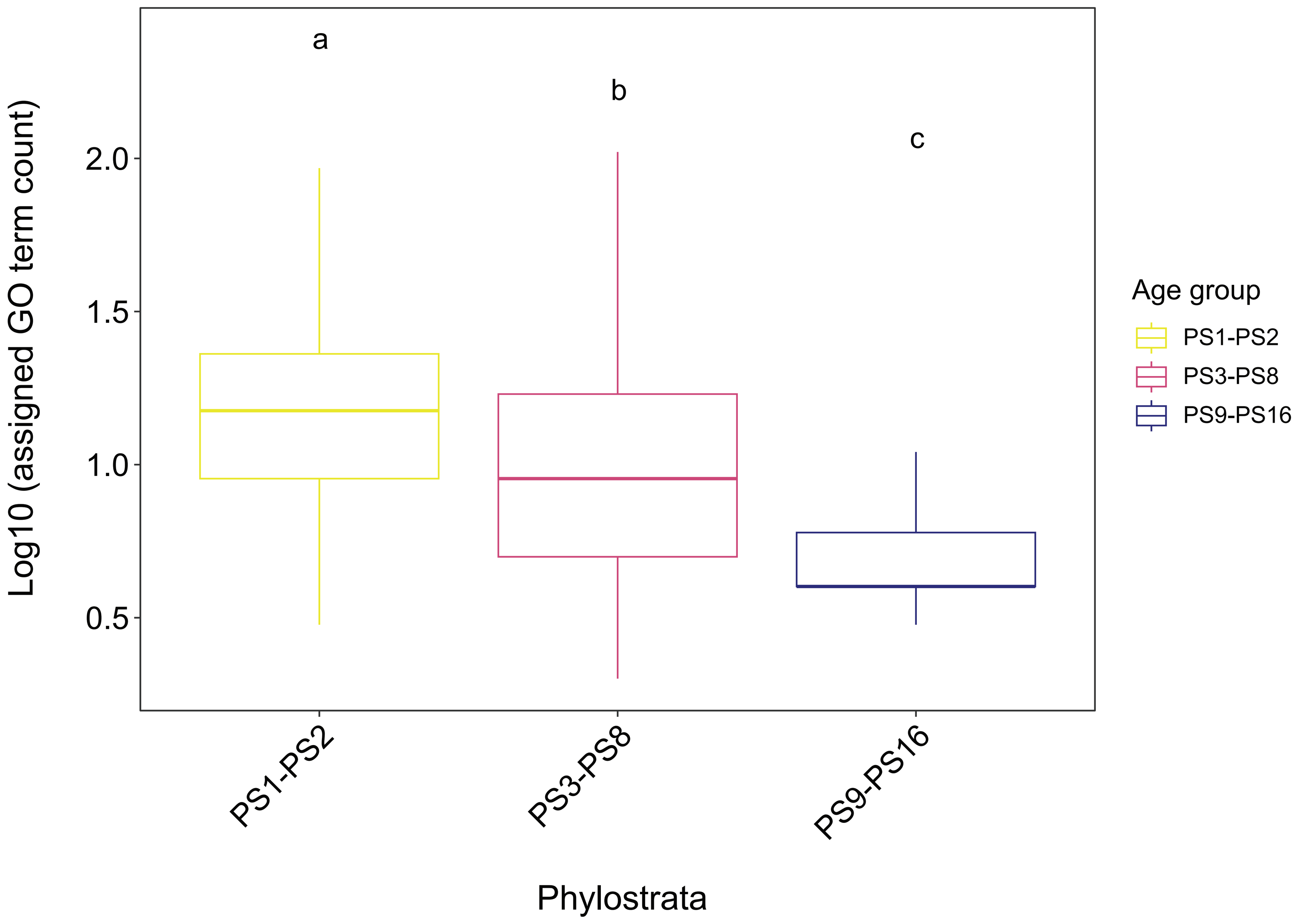

Supplementary Figure S9. Number of GO annotation terms assigned to Arabidopsis genes from different phylostrata groups. Younger phylostrata groups (PS3-PS8 and PS9-PS16) have significantly less annotation terms compared to older phylostrata groups (PS1-PS2). Different letters indicate groups that are significantly different based on Dunn's test followed by post hoc analysis. Horizontal line in the middle of the boxes represent the group mean. Upper and lower edges of each box represent the 75th and 25th percentile, respectively. Whiskers represent the variability of the data within each group.

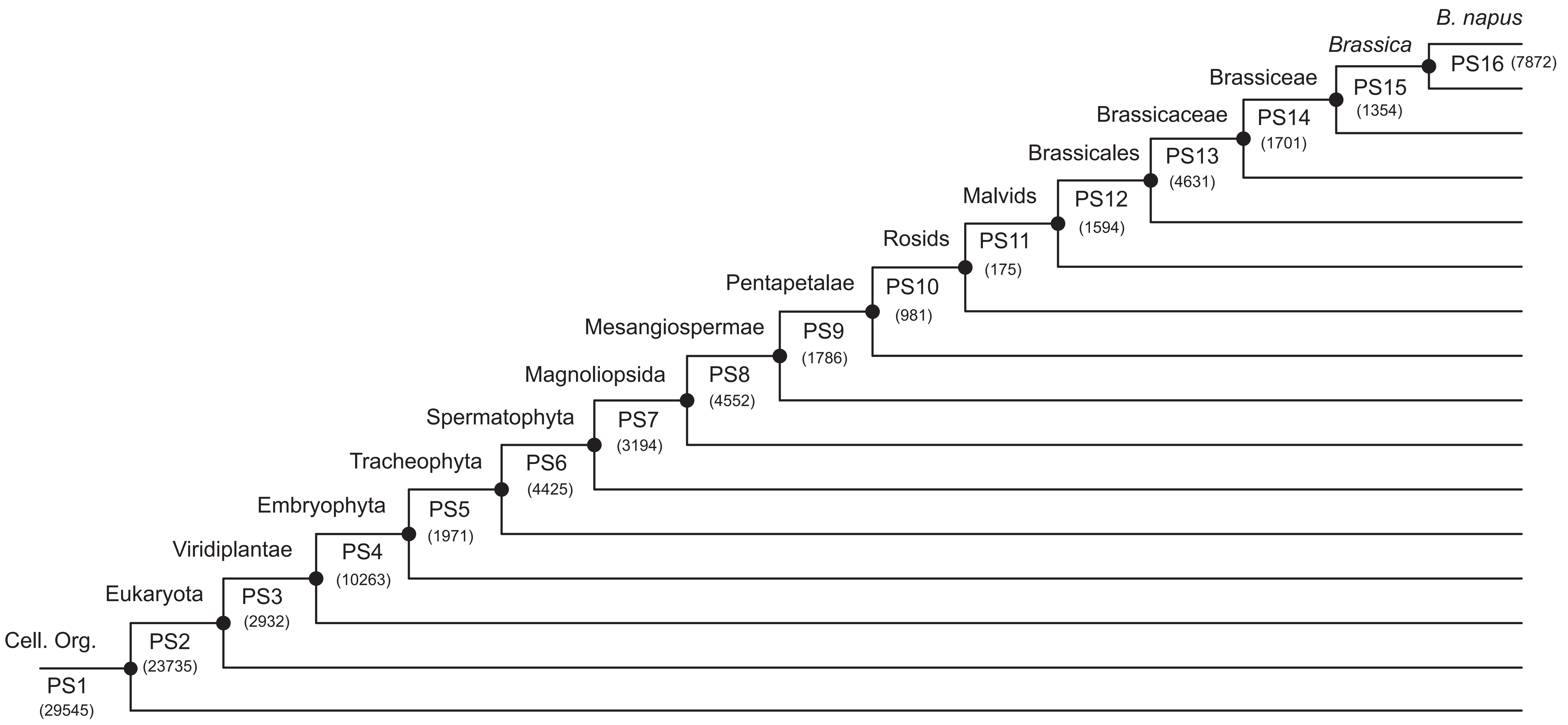

Supplementary Figure S10. Phylostratigraphy of *B. napus* genes. The number of genes under each phylostratum is indicated within brackets.

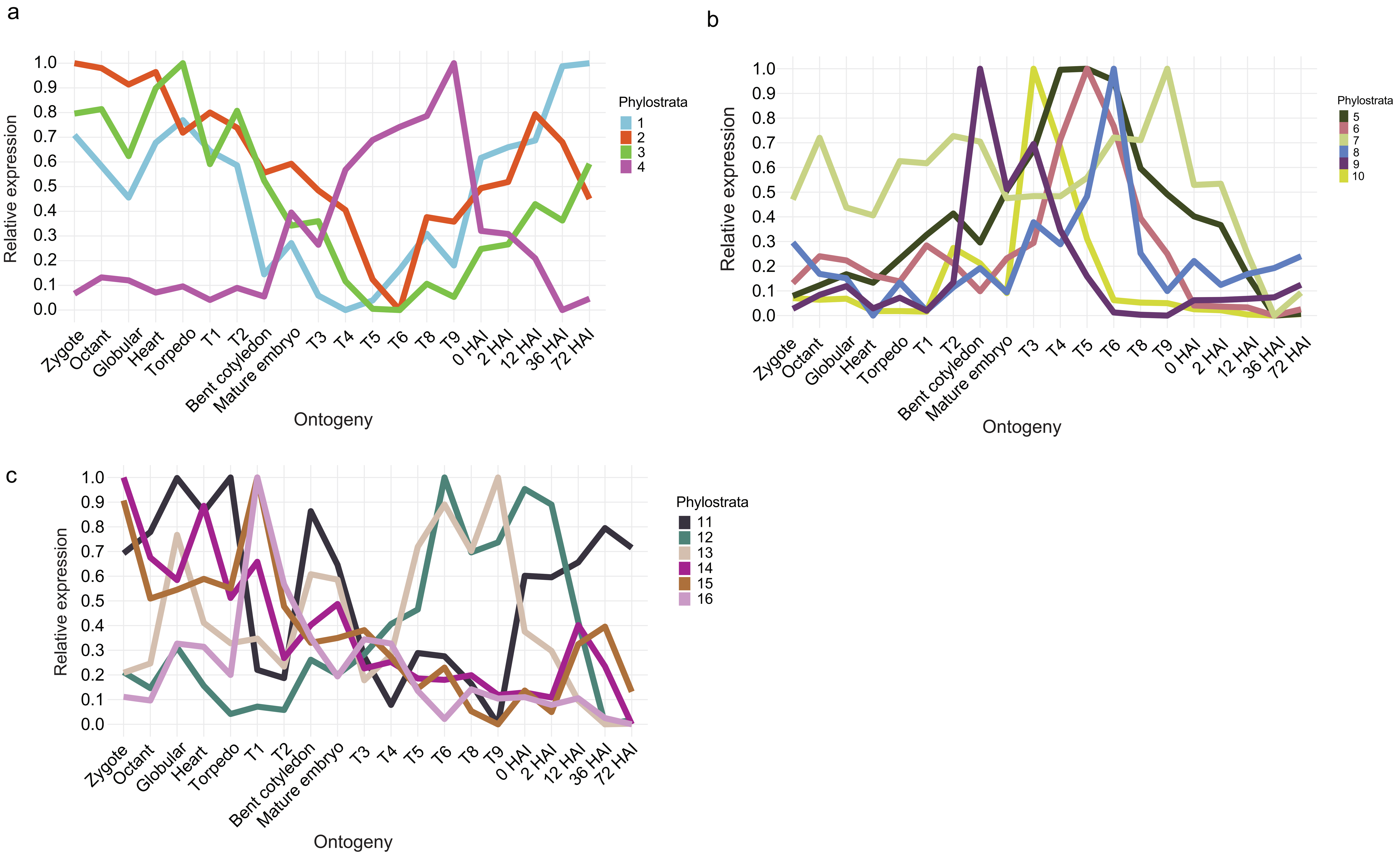

Supplementary Figure S11. Relative expression of each phylostrata during *B. napus* seed life cycle. **a**, PS1-PS4; **b**, PS5-PS10; and **c**, PS11-PS16.

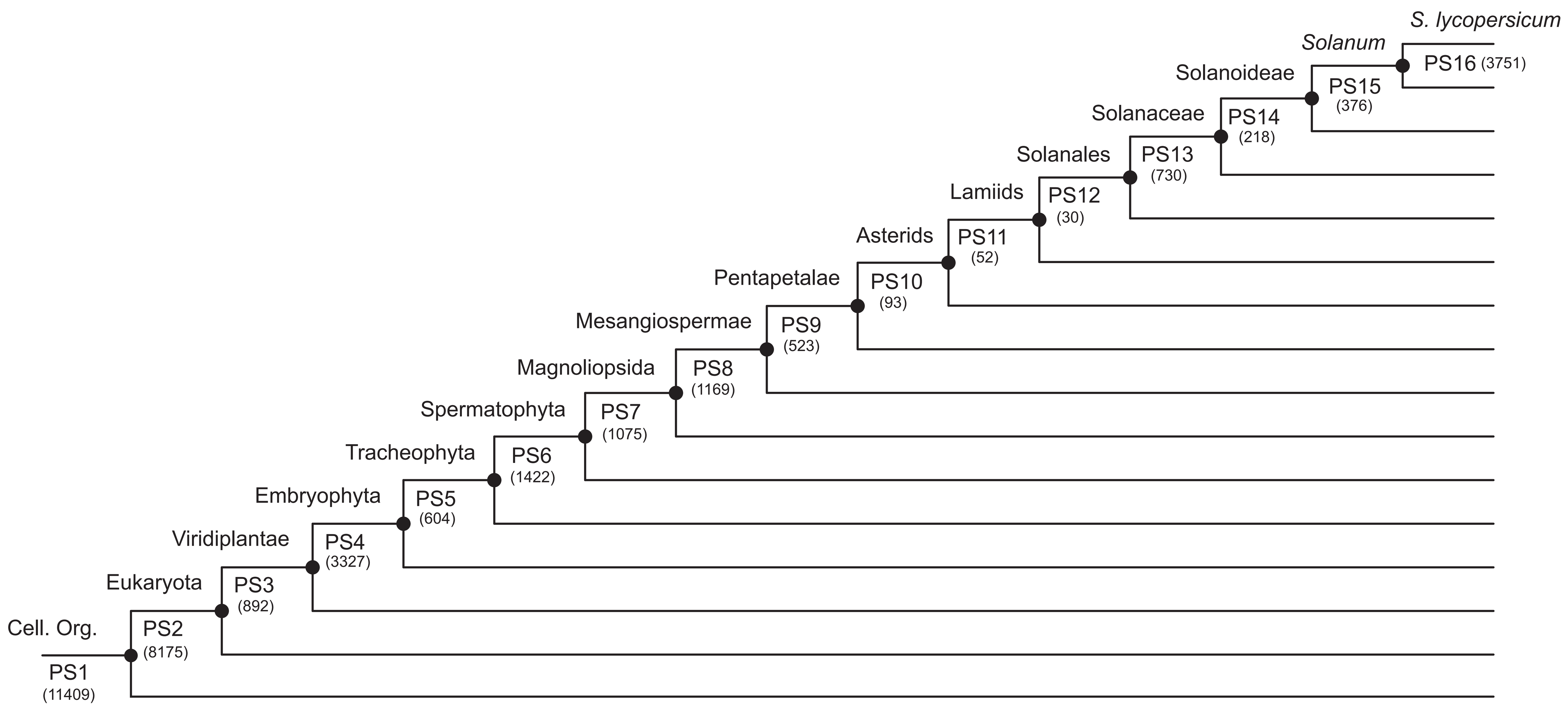

Supplementary Figure S12. Phylostratigraphy of *S. lycopersicum* genes. The number of genes under each phylostratum is indicated within brackets.

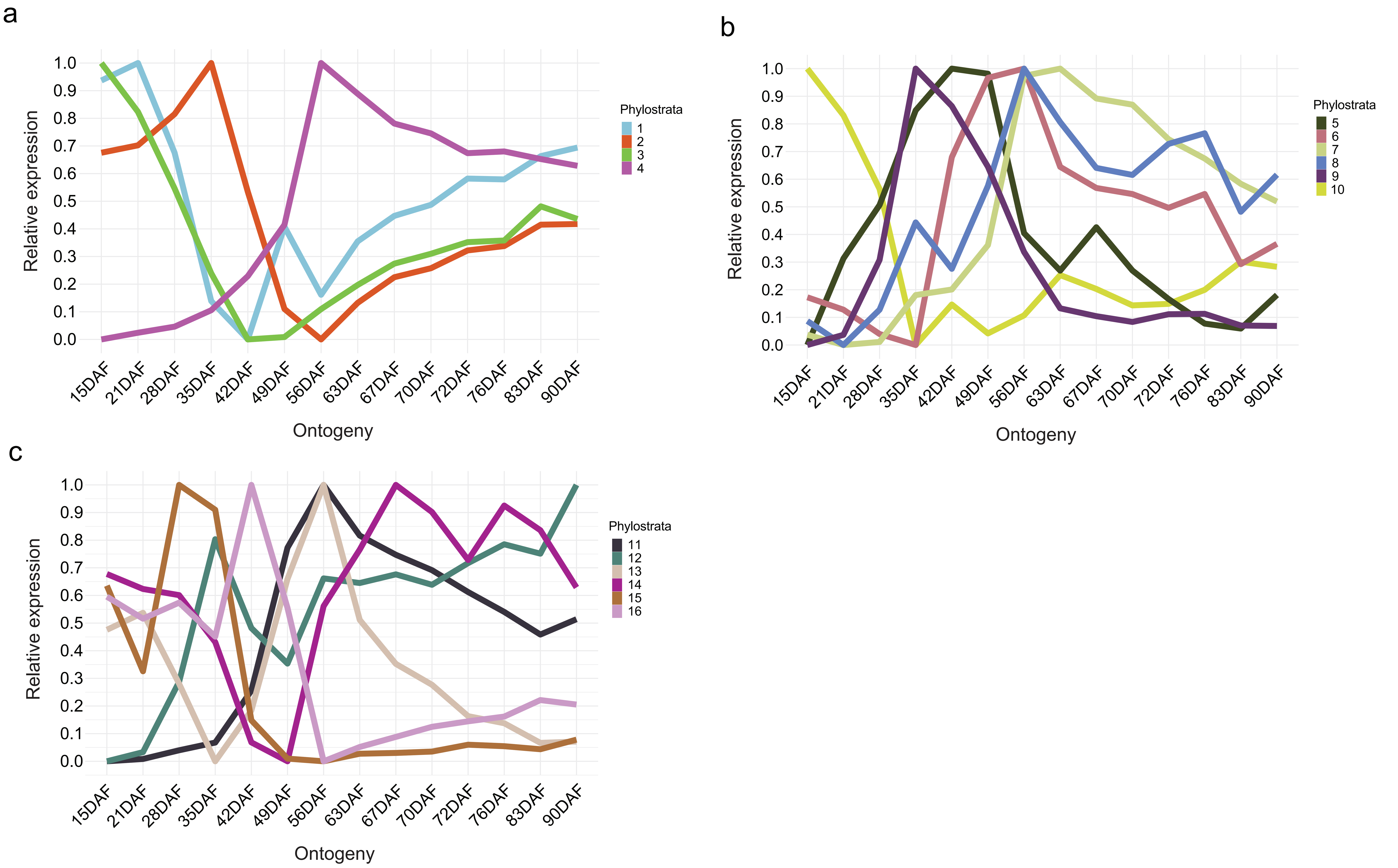

Supplementary Figure S13. Relative expression of individual phylostrata during part of *S. lycopersicum* seed life cycle. a, PS1-PS4; b, PS5-PS10; and c, PS11-PS16. DAF indicates days after flowering.

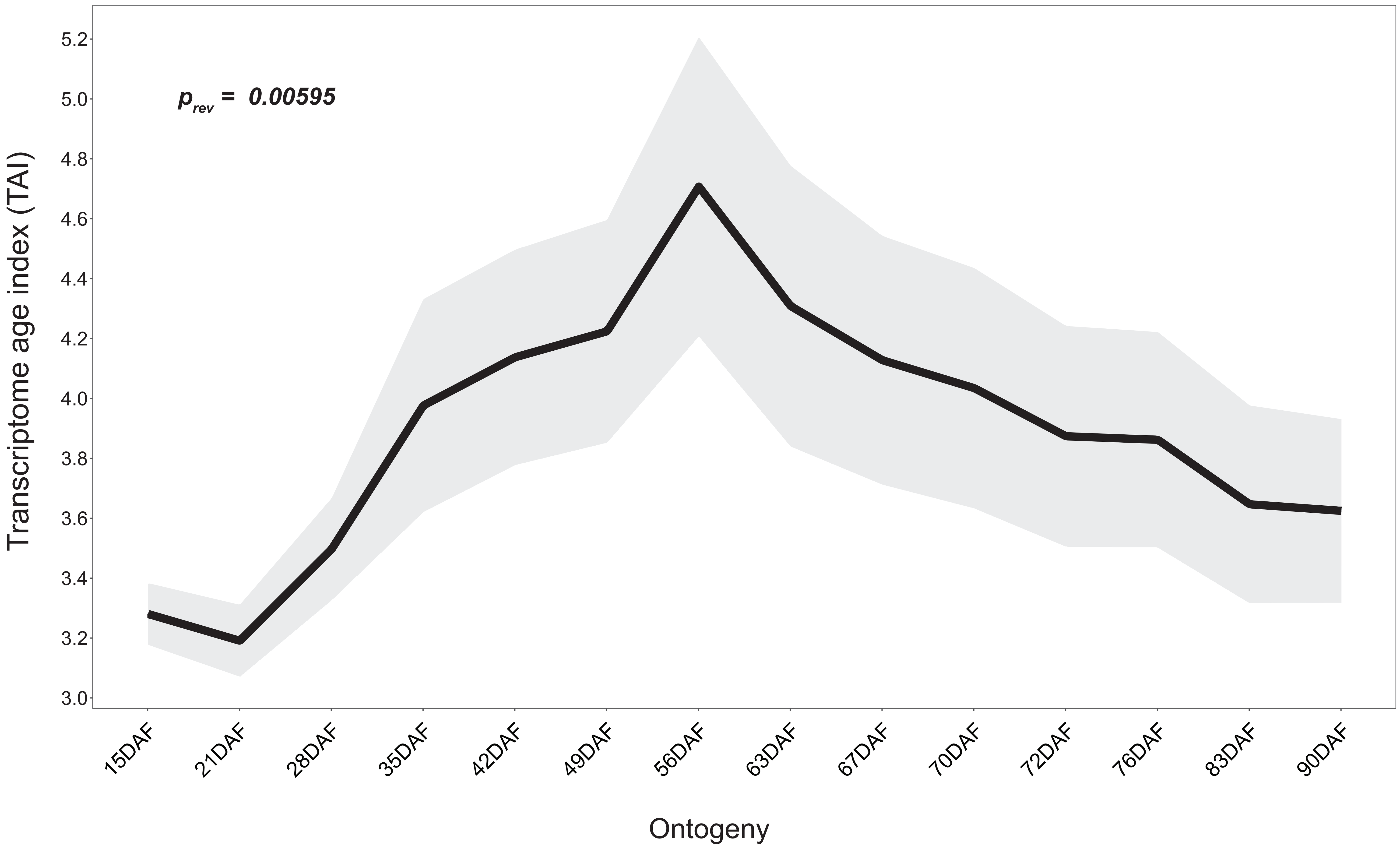

Supplementary Figure S14. TAI profile during *S. lycopersicum* seed maturation excluding genes from PS16. Grey area indicates the standard deviation.

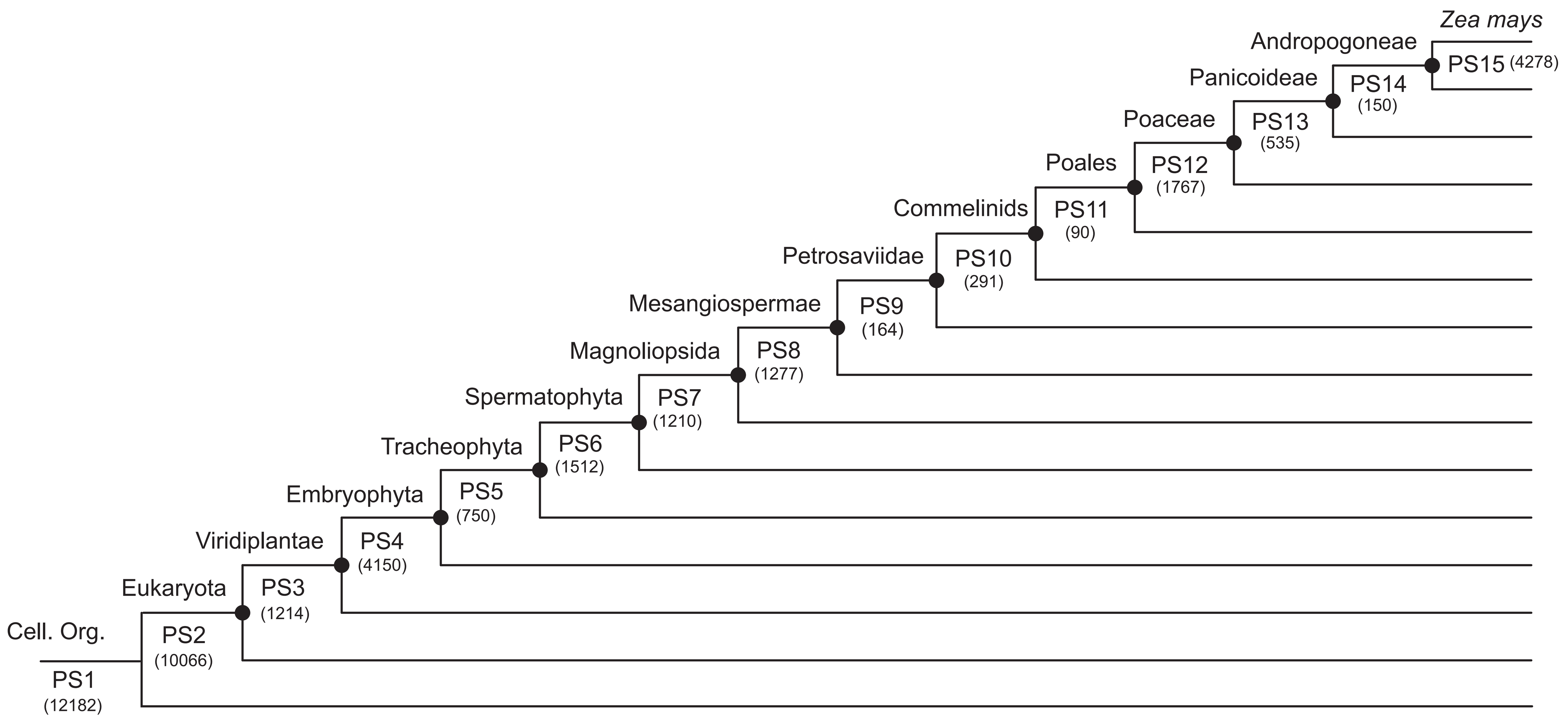

Supplementary Figure S15. Phylostratigraphy of *Z. mays* genes. The number of genes under each phylostratum is indicated within brackets.

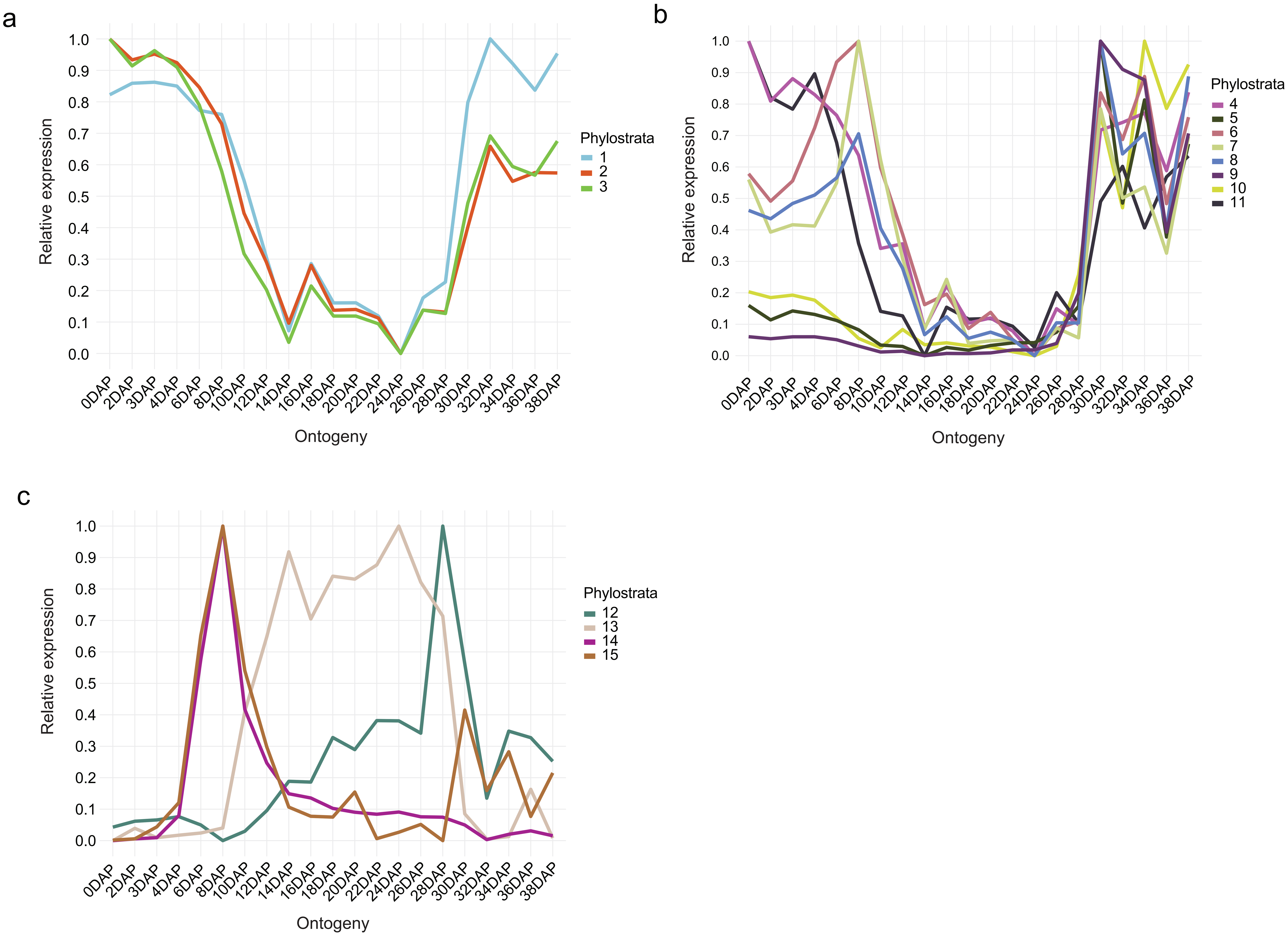

Supplementary Figure S16. Relative expression of individual phylostrata during *Z. mays* seed life cycle. a, PS1-PS3; b, PS4-PS11; and c, PS12-PS15.

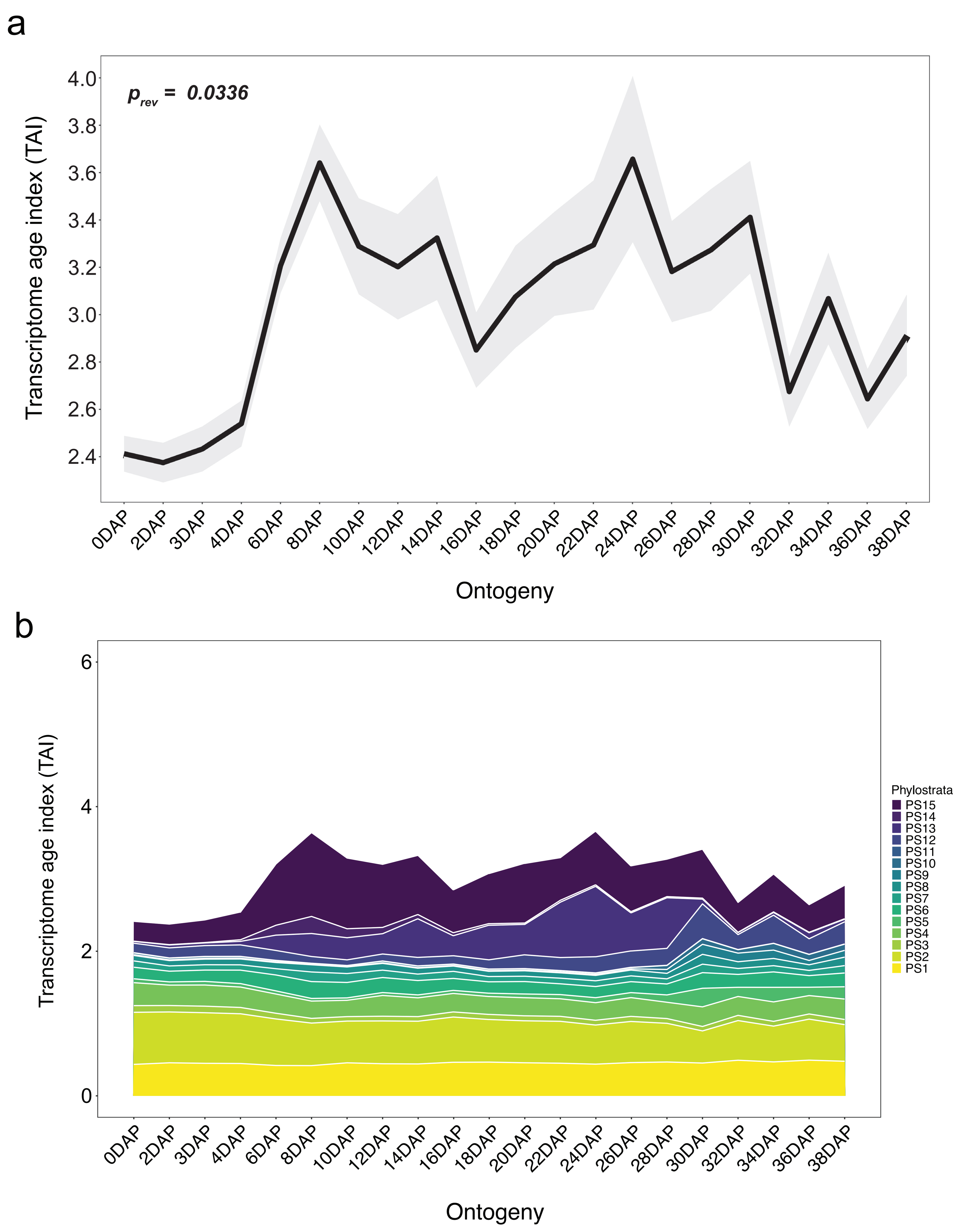

Supplementary Figure S17. Phylotranscriptomics pattern of *Z. mays* without zein genes. **a**, TAI pattern over parts of the seed life cycle without zein genes. Grey area indicates the standard deviation. **b**, Individual phylostrata contribution to TAI profile without zein genes.

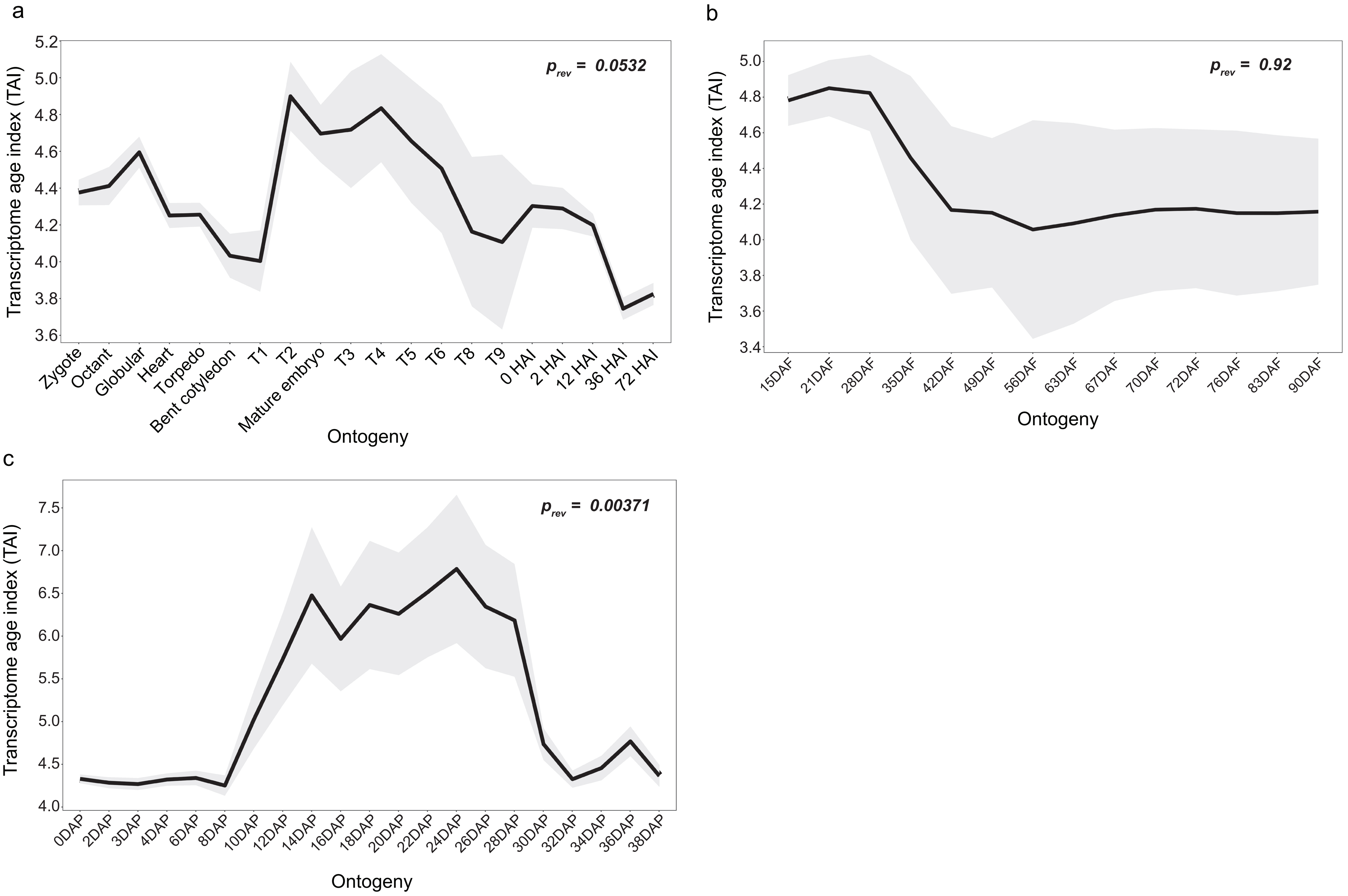

Supplementary Figure S18. TDI pattern during parts of the seed life cycle in three angiosperm species - **a**, *B. napus*; **b**, *S. lycopersicum*; and **c**, *Z. mays*. The p-value was significant for a reverse hourglass test in *B. napus* and *Z. mays* but not in *S. lycopersicum.* Grey area indicates the standard deviation.

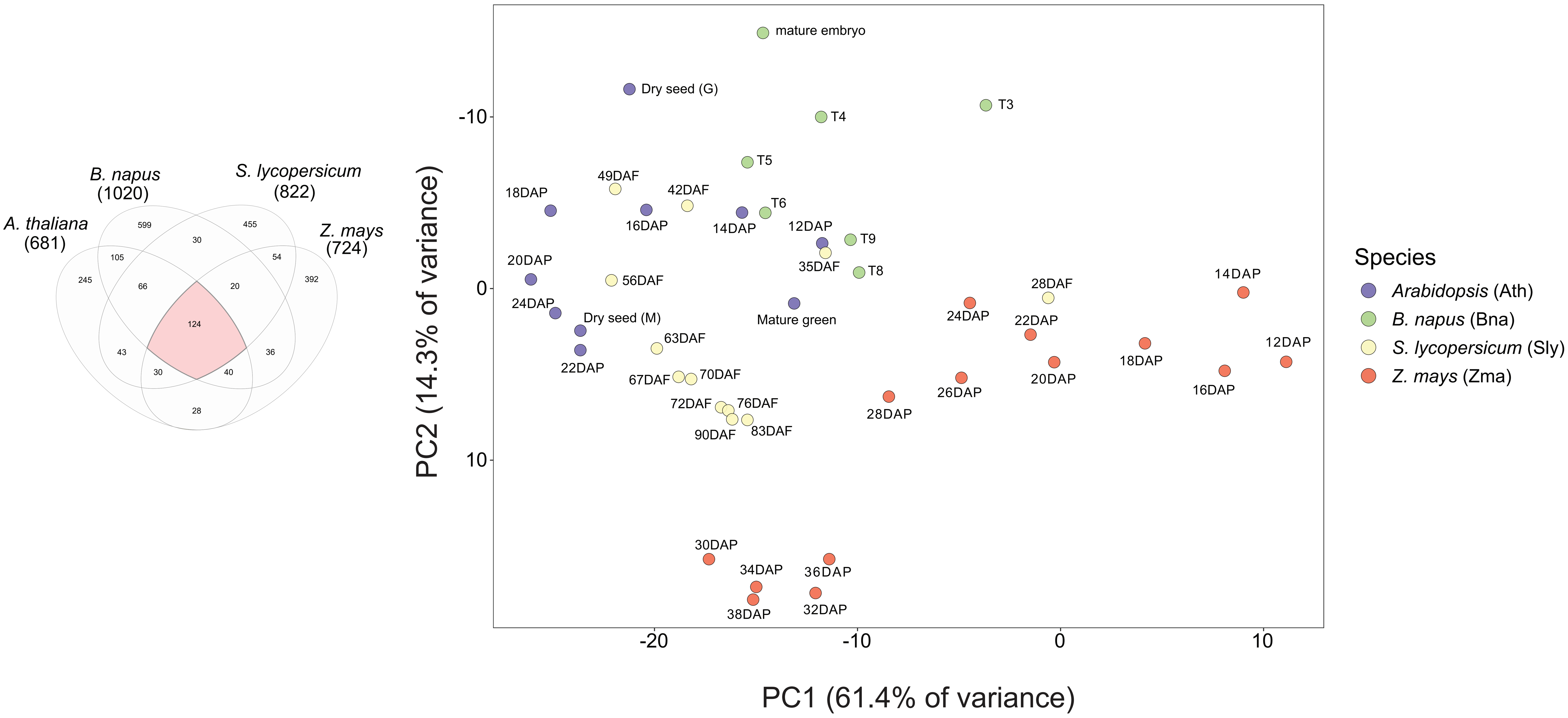

Supplementary Figure S19. PCA based on the 124 shared top 5% maturation orthogroups (red area in Venn diagram). Time points from only the maturation phase from all four species were used for the PCA.

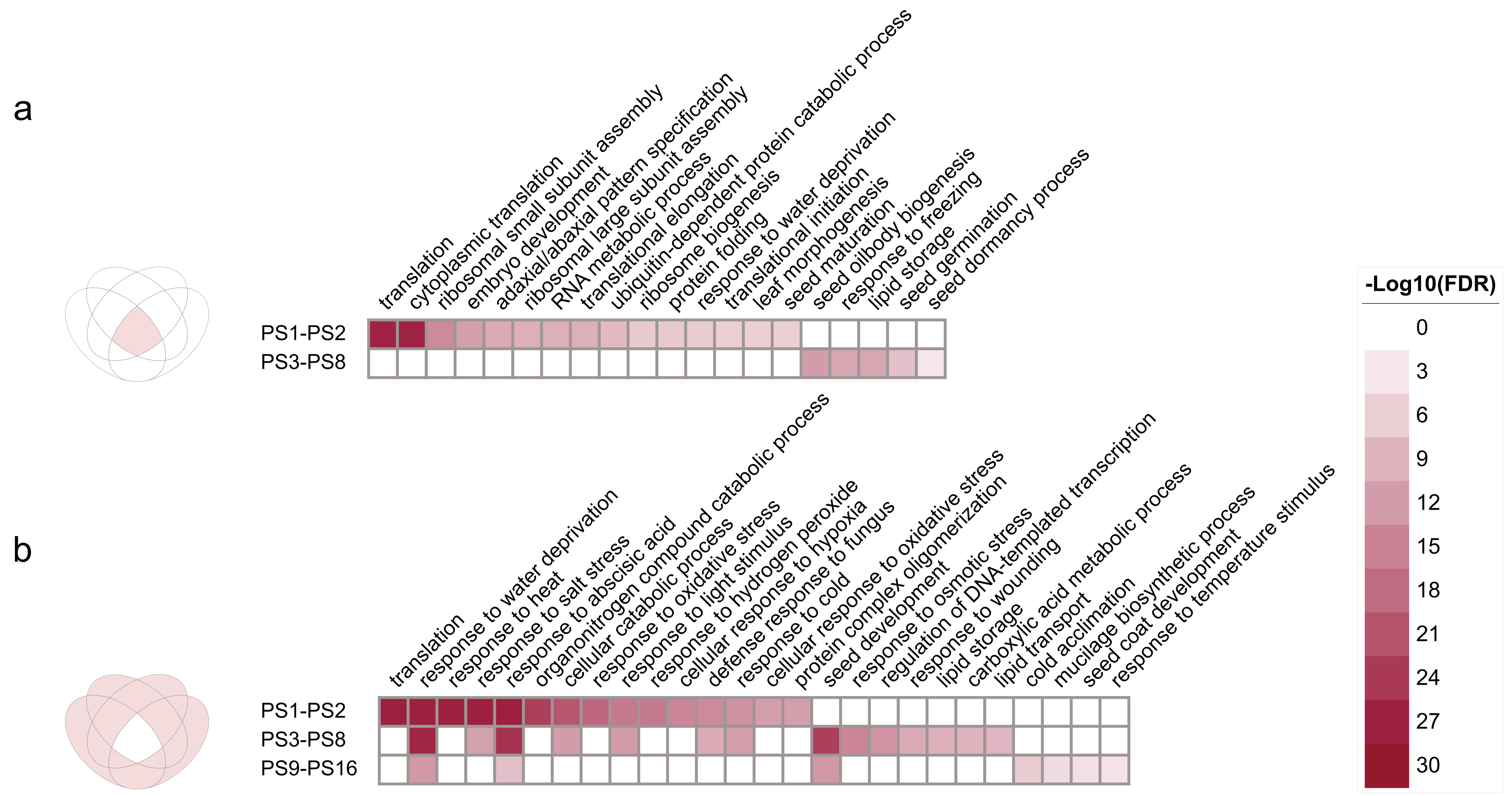

Supplementary Figure S20. Top GO terms (sorted based on -Log_{10}(FDR)) enriched in Arabidopsis genes that belong to **a**, conserved top 5% orthogroups; and **b**, non-conserved orthogroups.

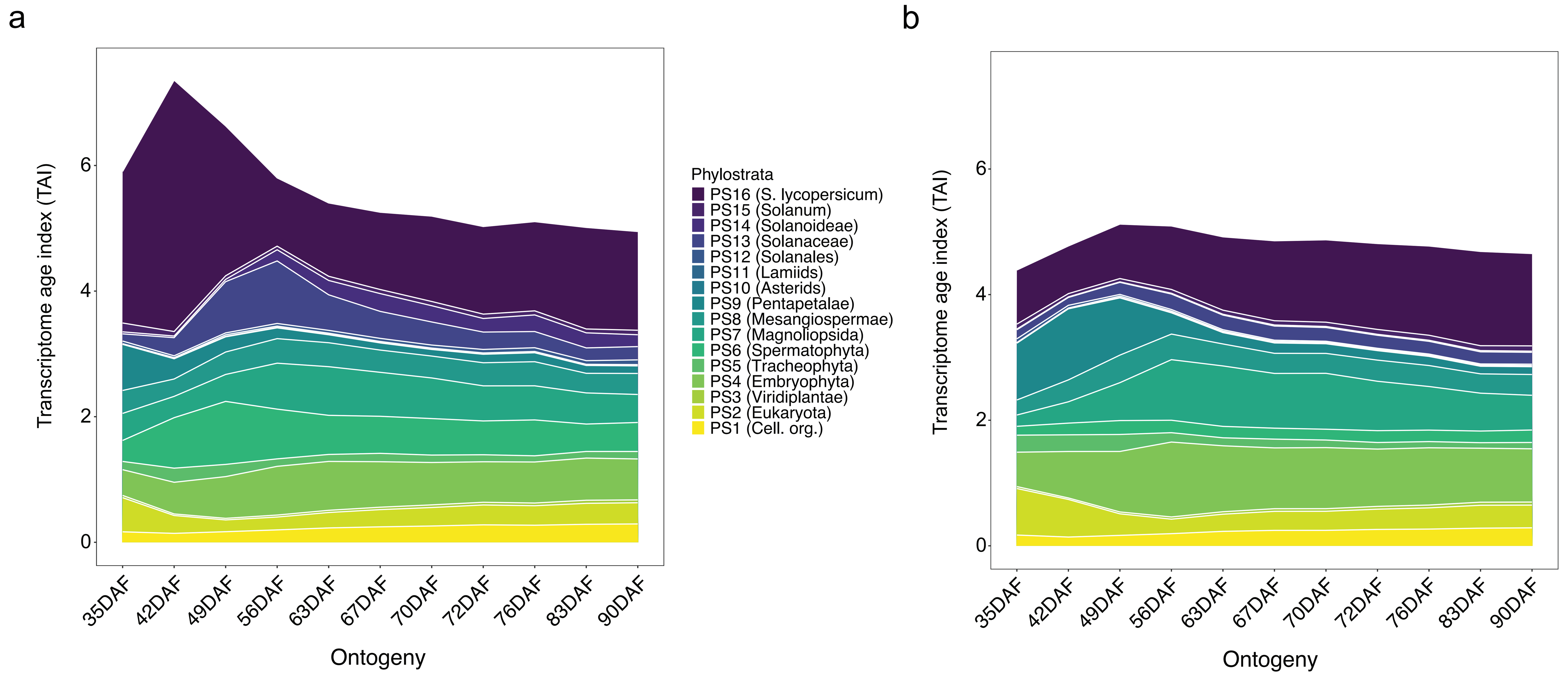

Supplementary Figure S21. Tissue-specific individual phylostrata contribution to the overall observed TAI profile in *S. lycopersicum*. **a**, endosperm TAI; and **b**, embryo TAI.

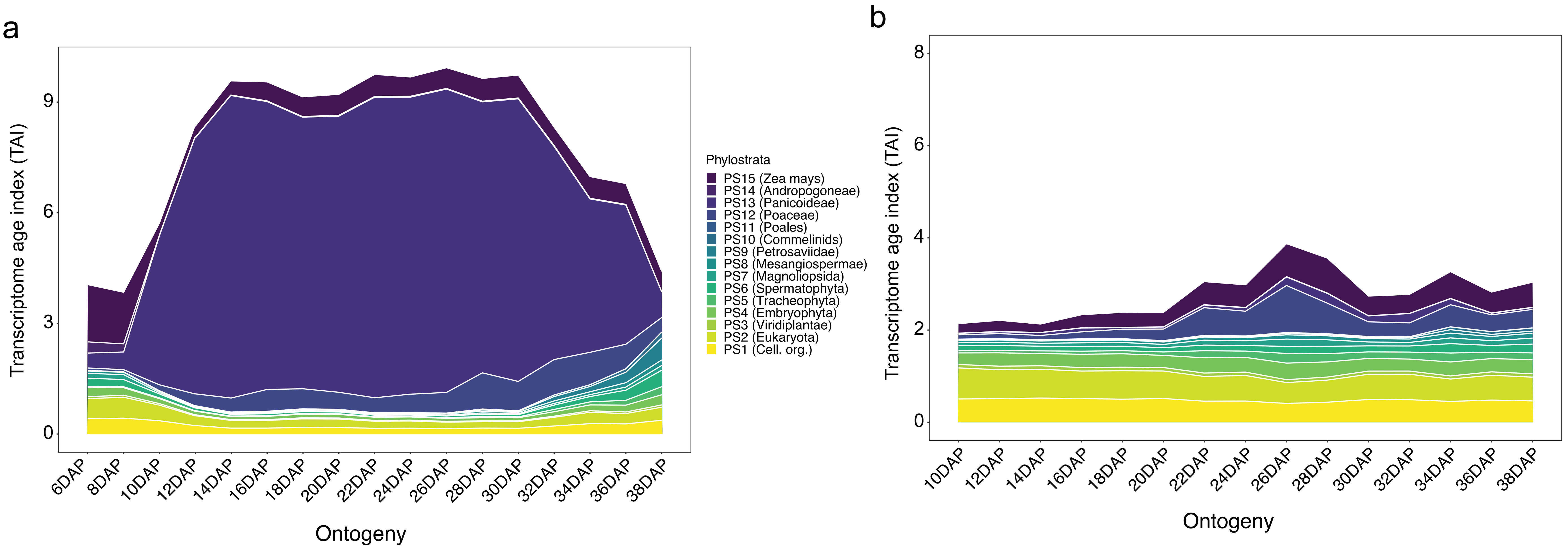

Supplementary Figure S22. Tissue-specific individual phylostrata contribution to the overall observed TAI profile in *Z. mays*. **a**, endosperm TAI; and **b**, embryo TAI.

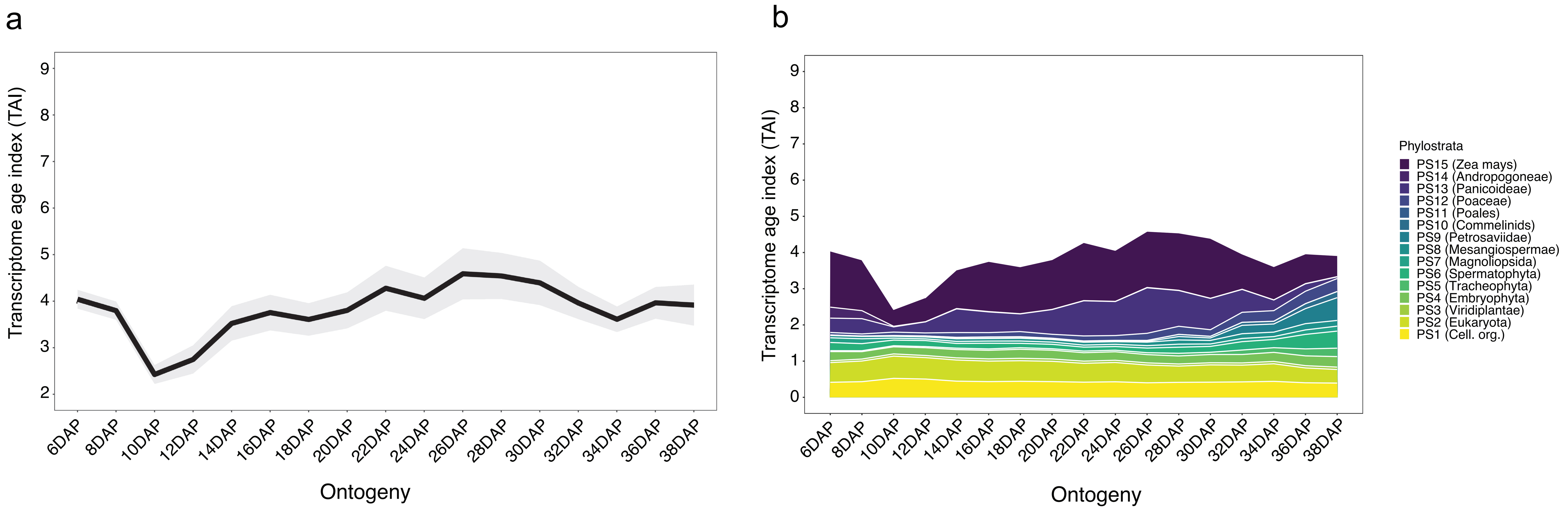

Supplementary Figure S23. TAI profile of *Z. mays* endosperm tissue without zein genes. **a,** TAI profile with the grey area indicates the standard deviation. **b,** Contribution of each phylostratum.

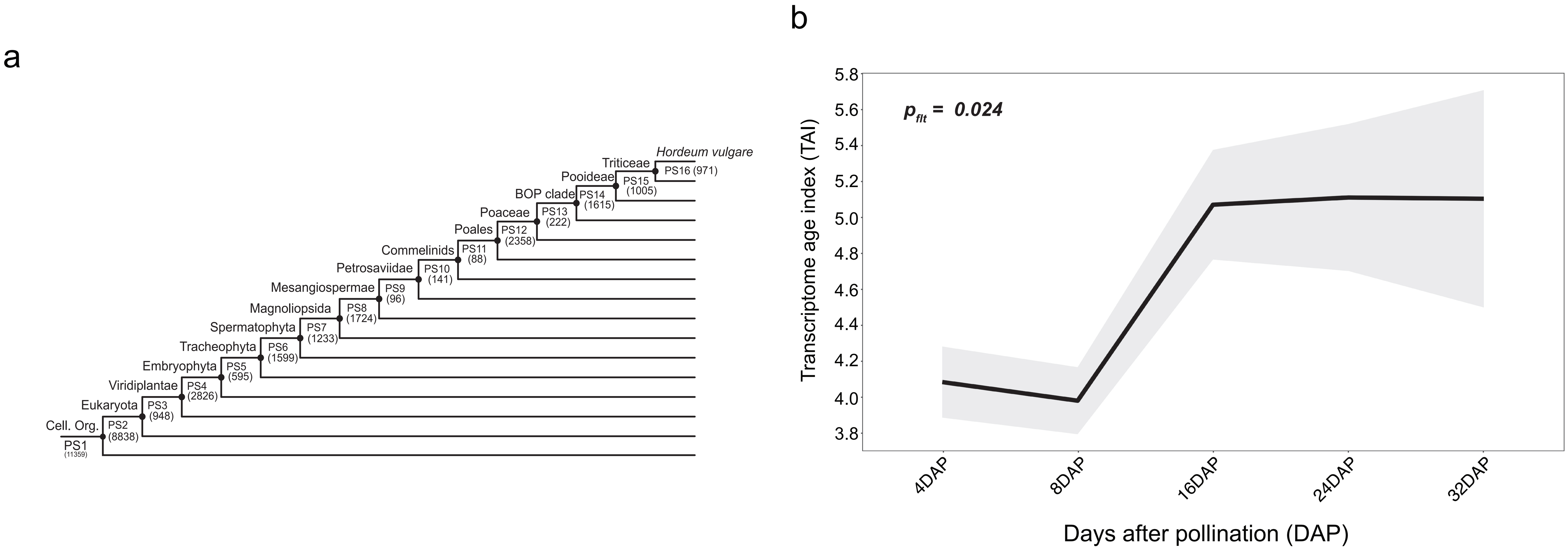

Supplementary Figure S24. Phylotranscriptome of parts of *H. vulgare* seed life cycle. **a**, Phylostratigraphy of *H. vulgare* genes. The number of genes under each phylostratum is indicated within brackets. **b**, TAI profile during part of *H. vulgare* seed life cycle. Grey area indicates the standard deviation.

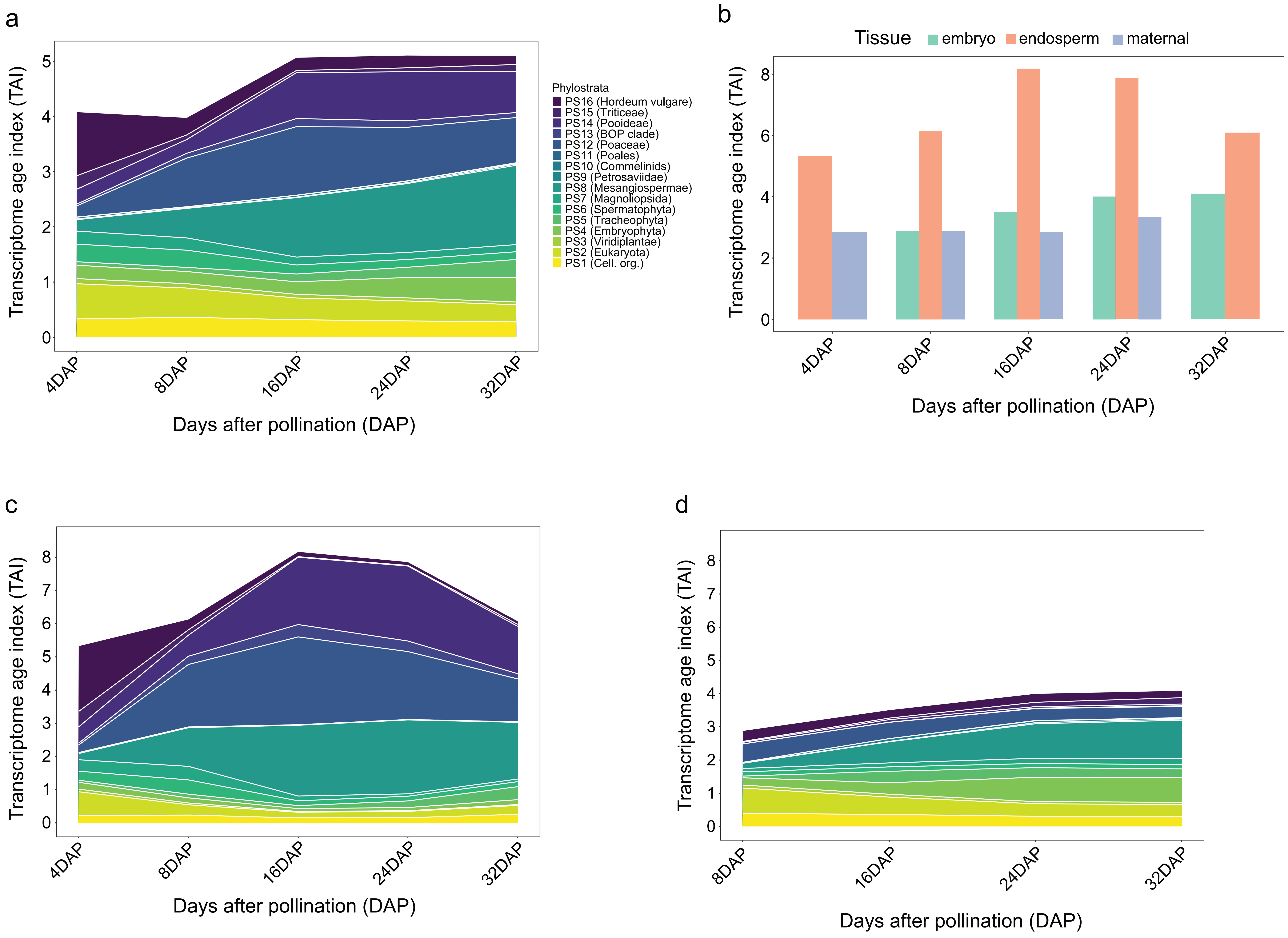

Supplementary Figure S25. Contribution of individual phylostrata to the TAI profile of *H. vulgare* during part of the seed life cycle. **a**, TAI profile of the whole seed tissue. b, Distribution of TAI values across the different tissues. **c**, TAI profile of endosperm tissue. **d**, TAI profile of embryo tissue. This is consistent with endosperm TAI contribution seen in Figure 4c for *Z. mays*.

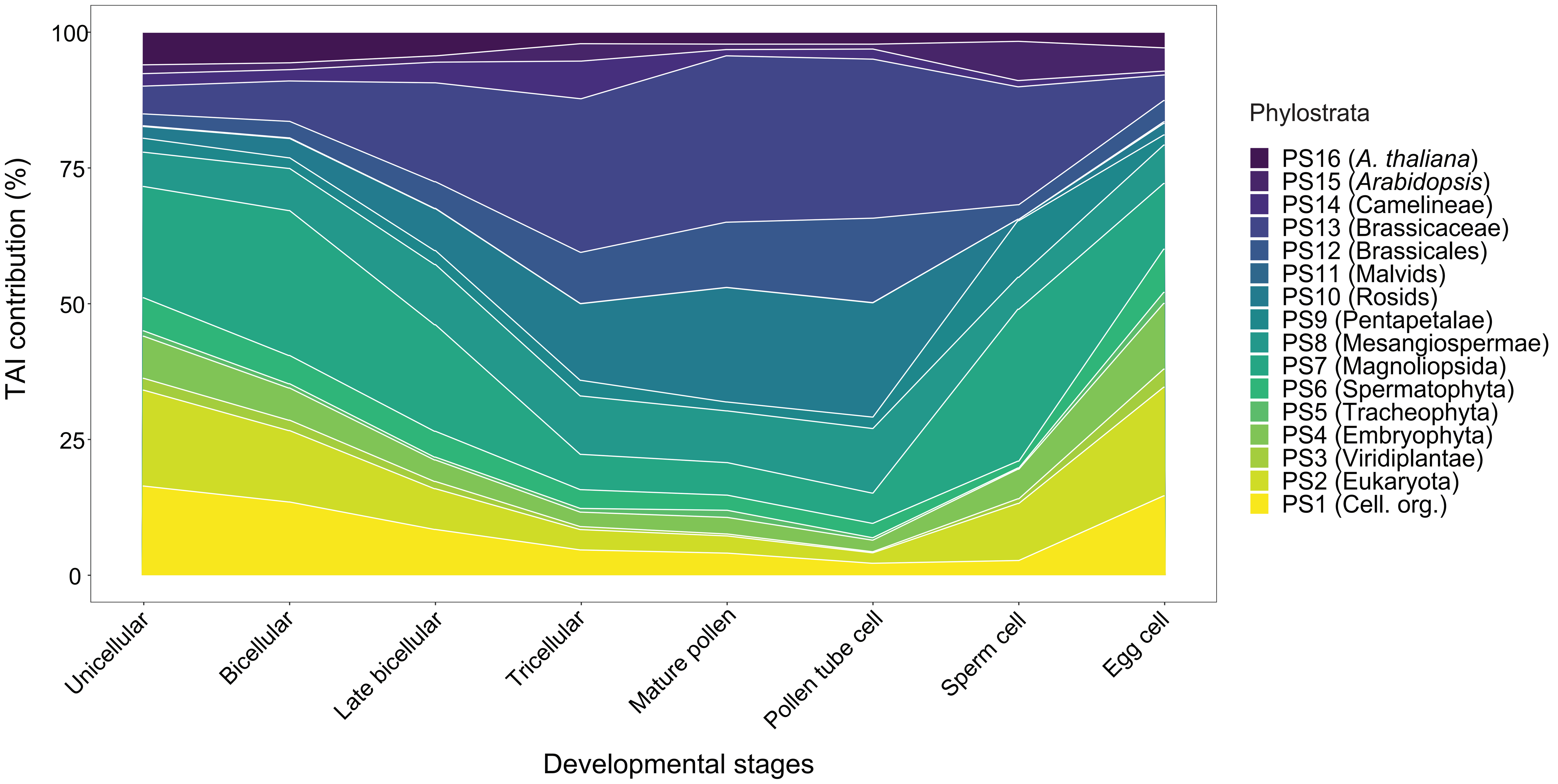

Supplementary Figure S26. Percentage contribution of each phylostrata (PS1-PS16) to the overall TAI profile during Arabidopsis pollen development and egg cell stage. Similar to Figure 5b, this shows that younger phylostrata genes have a higher contribution to TAI during pollen development.

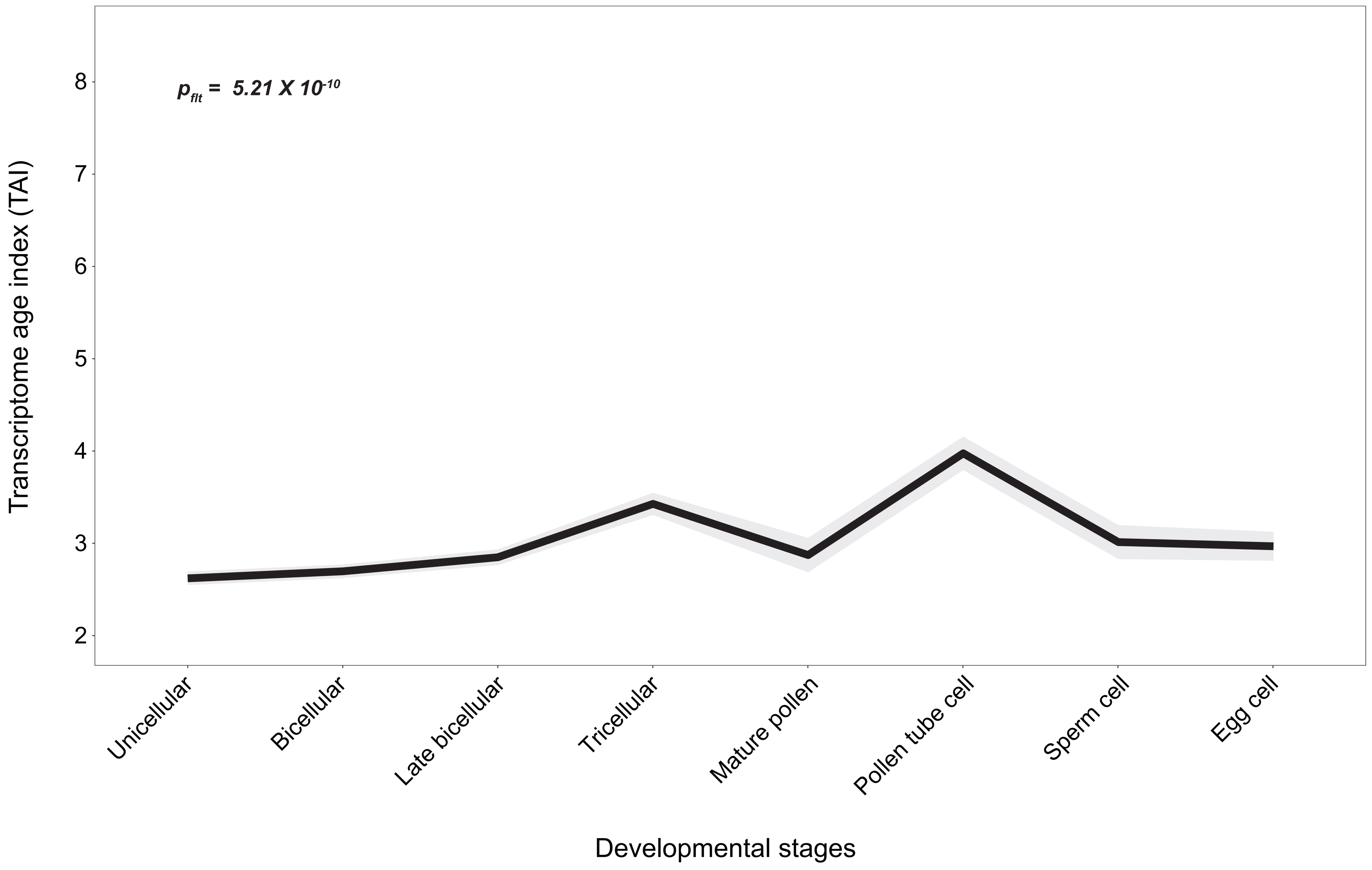

Supplementary Figure S27. Arabidopsis pollen TAI profile without top 5% pollen expressed genes. Grey area indicates the standard deviation.

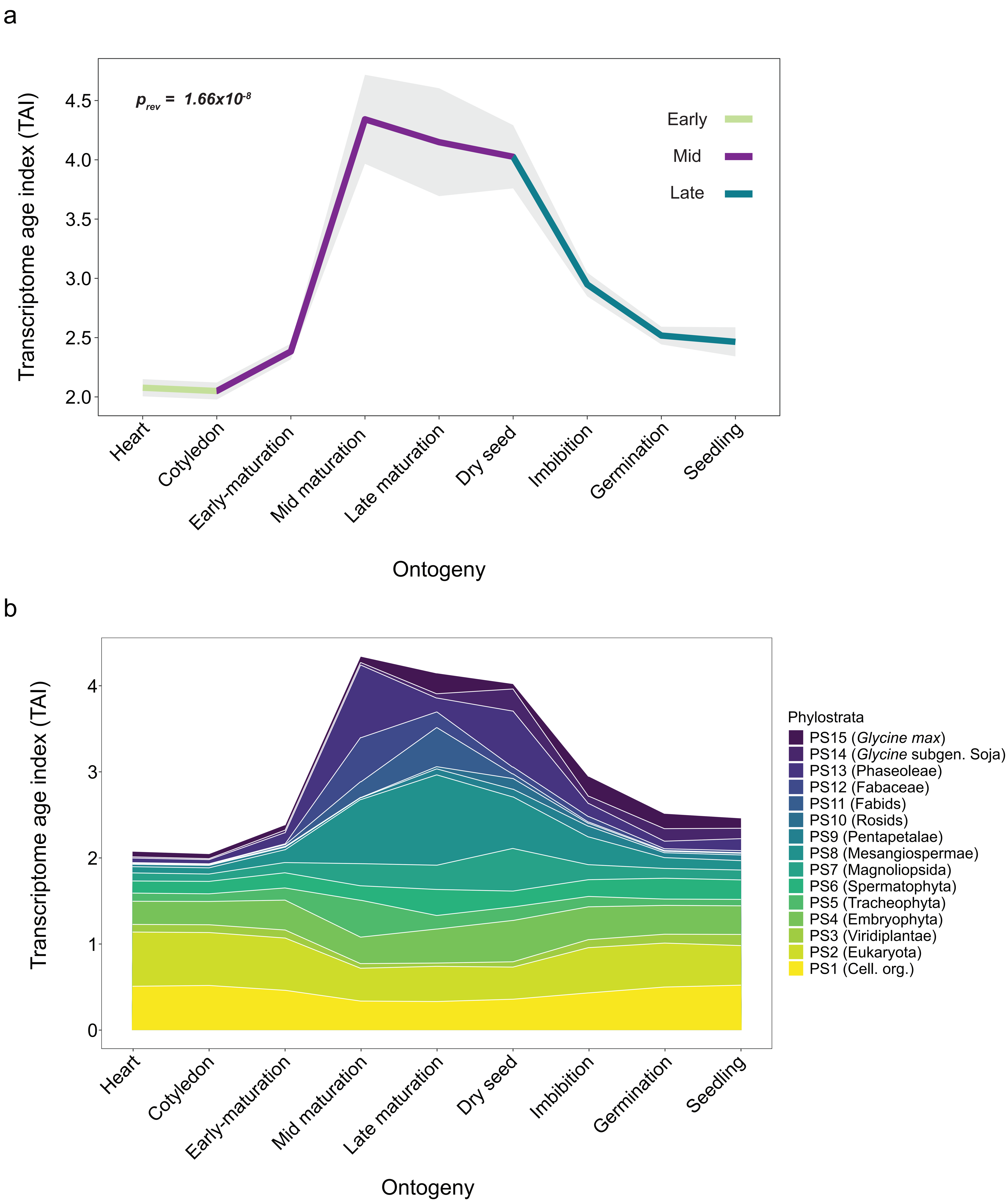

Supplementary Figure S28. a, TAI profile during the entire seed life cycle in soybean (*Glycine max*) (data from Chen et al. 2024). Colored segments of the line indicate stages that were considered part of early, mid, and late development, respectively, while performing the reverse hourglass test. The p-value indicates the significance of the test. Grey area indicates the standard deviation. **b**, Contribution of individual phylosrata to the overall TAI profile in soybean.
